# Assessing the impact of the president’s emergency plan for AIDS relief on all-cause mortality

**DOI:** 10.1371/journal.pgph.0002467

**Published:** 2024-01-18

**Authors:** Gary Gaumer, Yiqun Luan, Dhwani Hariharan, William Crown, Jennifer Kates, Monica Jordan, Clare L. Hurley, Allyala Nandakumar

**Affiliations:** 1 Institute for Global Health and Development, The Heller School for Social Policy and Management, Brandeis University, Waltham, Massachusetts, United States of America; 2 Global Health & HIV Policy Program, KFF, Washington, District of Columbia, United States of America; Johns Hopkins University Bloomberg School of Public Health, COLOMBIA

## Abstract

This study estimated the impacts of PEPFAR on all-cause mortality (ACM) rates (deaths per 1,000 population) across PEPFAR recipient countries from 2004–2018. As PEPFAR moves into its 3rd decade, this study supplements the existing literature on PEPFAR ‘s overall effectiveness in saving lives by focusing impact estimates on the important subgroups of countries that received different intensities of aid, and provides estimates of impact for different phases of this 15-year period study. The study uses a country-level panel data set of 157 low- and middle-income countries (LMICs) from 1990–2018, including 90 PEPFAR recipient countries receiving bilateral aid from the U.S. government, employing difference-in-differences (DID) econometric models with several model specifications, including models with differing baseline covariates, and models with yearly covariates including other donor spending and domestic health spending. Using five different model specifications, a 10–21% decline in ACM rates from 2004 to 2018 is attributed to PEPFAR presence in the group of 90 recipient countries. Declines are somewhat larger (15–25%) in those countries that are subject to PEPFAR’s country operational planning (COP) process, and where PEPFAR per capita aid amounts are largest (17–27%). Across the 90 recipient countries we study, the average impact across models is estimated to be a 7.6% reduction in ACM in the first 5-year period (2004–2008), somewhat smaller in the second 5-year period (5.5%) and in the third 5-year period (4.7%). In COP countries the impacts show decreases in ACM of 7.4% in the first period attributed to PEPFAR, 7.7% reductions in the second, and 6.6% reductions in the third. PEPFAR presence is correlated with large declines in the ACM rate, and the overall life-saving results persisted over time. The effects of PEFAR on ACM have been large, suggesting the possibility of spillover life-saving impacts of PEPFAR programming beyond HIV disease alone.

## Introduction

The U.S. government, through the President’s Emergency Plan for AIDS Relief (PEPFAR), committed approximately $70 billion to address HIV/AIDS in low- and middle-income countries (LMICs) through bilateral efforts between 2004 and 2018 [1]. Services supported include the costs of antiretroviral therapy (ART), care and support for families, HIV testing, prevention services, voluntary male medical circumcision, and other services such as health worker training, operations management, and health system strengthening [2]. Initially, the PEPFAR program led to quick implementation using large, primarily vertical programs in a selected group of high need, “focus” countries, a group that has expanded over time. While PEPFAR has provided bilateral funding to more than 100 countries over the 2004–2018 period, funding has been concentrated in a smaller subset of approximately 30 high-need countries, mostly in Africa. This subset of countries is subjected to the annual preparation of operating plans, performance monitoring, and budget negotiations [3]. PEPFAR funding increased quickly in its initial years, from 2004 to 2010, and then largely plateaued through 2018.

Research evidence has previously demonstrated that PEPFAR and other external health aid programs have saved millions of lives during the HIV epidemic. Early single-country studies of PEPFAR and other donor aid have documented substantial life-saving impacts including declines in all-cause mortality and increased life expectancy [4–6]. Multi-country PEPFAR impact studies evaluated PEPFAR “focus” countries, or PEPFAR recipients stratified by level of PEPFAR investment, and reported declines in the number of HIV-related deaths after 2003 [7–9]. Most recently a PEPFAR impact study [10] used panel data and a difference‐in‐difference (DID) design to assess PEPFAR’s impact on women and children in both “focus” and “non-focus” countries. That study found that funding from PEPFAR was associated with reductions in new HIV infections and HIV-related deaths among both women and children. Another study [11] found that between 2004 and 2013, 2.9 million HIV infections were averted with 11.6 million life years gained, and 9 million children were saved from becoming orphans.

In the largest and most thorough field evaluation of PEPFAR, the National Academy of Sciences [12] reviewed literature and program records, and conducted extensive field interviews, and concluded that PEPFAR made significant contributions in scaling up testing, counseling, and a variety of prevention and treatment programs, in addition to saving lives and improving the quality of life for persons living with HIV(PLHIV) [12].

In this study, we seek to add to the body of knowledge of PEPFAR’s impact by providing an assessment over 15 years of the program, from 2004–2018, across subgroups of recipient countries and over separate programmatic phases.

We examine three overall research questions:

1. Over the 2004–2018 period, was PEPFAR’s presence in low-and middle-income countries (LMICs) associated with greater reductions in all-cause mortality (ACM) than would have been expected in the absence of PEPFAR?
2. Were there differences in the impact of PEPFAR across different phases of the 15-year period used in this assessment?
3. Are there variations in the impact of PEPFAR across countries arising from differences in the intensity of PEPFAR financial support, and/or due to the participation in the COP process involving careful planning and monitoring?

## Methods

We assessed the impact of PEPFAR presence on the ACM rate (deaths per 1,000) by analyzing a 29-year panel dataset (1990–2018) of 157 low- and middle-income countries that included 90 PEPFAR countries and 67 countries in the control group. The ACM rate was selected as our outcome of interest to overcome issues in the reliability of using an HIV-specific outcome measure and to capture possible spillover impacts beyond HIV mortality [13].

Data sources include the World Bank’s World Development Indicators (WDI) [14], the U.S. government’s foreignassistance.gov database [15], the OECD Creditor Reporting System database [16], the United Nations Department of Economic and Social Affairs [17], and the Institute of Health Metrics and Evaluation (IHME) GBD Result’s Tool [18].

### Cohorts

Our PEPFAR recipient group included 90 countries that had received at least $1 million in PEPFAR support over the 2004–2018 period. Our control group included 49 low- and middle-income countries that had not received any PEPFAR support and 18 LMICs that received minimal PEPFAR support (<$1M total or <$0.05 per capita cumulatively) between 2004 and 2018. Complete lists of countries and modeling results are provided in S1 Text and S1 Table Appendices. Ethics approval was not required for this study. Patients or the public were not involved in this research study.

In addition to examining outcomes for all PEPFAR recipient countries taken together, we also estimated PEPFAR impacts on separate cohorts among the 90 recipient countries. These separate cohorts included tertiles (three equally sized cohorts) of countries with the highest, medium, and lowest PEPFAR spending (cumulative PEPFAR disbursements per capita from 2004 to 2018). A second set of cohorts were countries that were subject to annual country-operating plans (COP), and those countries that did not have COP status. We also examined impacts separately for three different five-year time periods of program operation (2004–2008, 2004–2013, 2004–2018), to assess the size and direction of PEPFAR impacts over time. These periods generally correspond to the three authorizing periods for the program. S2 Text in the Appendix provides more details on how we created PEPFAR country cohorts for analysis.

### Difference-in-differences models and covariates

Difference-in-differences (DID) modeling is used to obtain PEPFAR impact estimates. DID controls for any unobserved differences between intervention and control groups that remain constant over time and is widely used in program evaluation research to provide program impact estimates [19] including previous evaluations of PEPFAR [7, 8]. The method can be used when pre and post data are available for countries that received PEPFAR funding and for those that did not (e.g. the control group). All of the DID models included three dummy variables: (1) a dummy variable that captures the overall differences in the mean value of the dependent variable between the baseline period (pre-2004) and the follow-up period. This measures the time trend in the control group; (2) a dummy variable for PEPFAR countries and control group countries that measures differences at baseline; and (3) an interaction dummy variable between the first two dummy variables, which is interpreted as the impact of the PEPFAR program. See more information on the difference-in-difference method in S3 Text.

To examine the consistency of impact estimates across alternative model specifications we estimated five models for each of our cohorts. Model 1 used no covariates other than the three DID dummy variables noted above. Model 2 included baseline (2004) values of population, GDP/capita, HIV prevalence rate, fertility rate, life expectancy at birth, percent population in urban areas, a dummy variable for whether the US provided HIV aid prior to 2004, a dummy variable for whether the country was a low-income or a middle-income country, and secondary school enrollment (% of gross). Model 3 added two additional baseline covariates: per capita other donor (non-PEPFAR) spending on health, and per capita domestic spending on health which is calculated as the aggregate of domestic government spending and private spending on health, in per capita term.

In models 1 through 3 our emphasis is on baseline covariates rather than time varying covariates, a deliberate decision made to avoid endogeneity problems. Causal inference regarding PEPFAR’s impact on all-cause mortality is derived using the Rubin potential outcomes framework [20]. This approach seeks to estimate the average treatment effect as the difference between the ACM outcomes for PEPFAR countries versus the outcomes that would have resulted had countries not been exposed to the PEPFAR program. The estimate of outcomes for the unexposed group of countries is derived from the control group. It is important that the control group be as similar as possible to the PEPFAR intervention group; balancing on baseline characteristics helps to achieve this. We estimate the causal parameter, the average treatment effect (ATE) of PEPFAR, using a differences-in-differences (DID) model.

Models 4 and 5 include yearly health spending covariates to control for potential confounding from donor and local policy activities. Model 4 has baseline covariates and a yearly variable for annual other donor health spending per capita. Model 5 adds (to model 4) yearly domestic health spending which is a combination of domestic government spending and private spending on health. The yearly health spending variables provide additional potential balancing of the PEPFAR and control group countries when estimating the impacts of PEPFAR. However, it is important to note that these variables could introduce endogeneity. In particular, changes in ACM and PEPFAR spending could also influence the annual spending variables added in models 4 and 5. For these, and other reasons, we conducted extensive specification testing on all of the models to better characterize their statistical robustness. We estimated logged and unlogged models for all five model specifications. These included the five main model specifications, each of which was estimated for cohort groups compared to the control group countries. We tested the normality of residuals for both the logged and unlogged models 4 and 5 using the Shapiro-Wilk test. The residuals for both sets of models were visually close to normal, although statistical testing revealed that they deviated from normality. The logged models were not, however, clearly superior in terms of normality and may introduce potential problems with retransformation bias when estimating program impacts in the presence of heteroscedasticity in the original ACM units. For this reason, we report the unlogged model results in this paper and provide the logged versions of the models in the S1 Table. Logged and unlogged models were highly consistent in terms of signs and statistical significance of the PEPFAR program intervention variable.

In models with time-varying covariates (models 4 and 5) we tested for potential endogeneity using the Hausman test. The results indicate that time varying variables were, indeed, endogenous. The time-varying variables included in Model 4 resulted in somewhat smaller estimates of PEPFAR impacts on ACM. It is not clear how much of this difference was due to the extra control introduced by the time-varying variables, endogeneity bias, or both. However, the results did not materially change the conclusion that PEPFAR has had very large and statistically significant, and persisting impacts on ACM throughout the 2004–2018 period. Only with Model 5 did we find results that suggested materially different policy conclusions than the models without time-varying covariates, although evidence of endogeneity suggests that these results should be interpreted with caution.

Finally, the DID parallel trends assumption was tested for all models and found to be upheld. All model results and specification testing are reported in the S1 Table, S1 Fig, and Fig A in S2 Fig.

Adjusted R-square values for model 1 are 0.10–0.20, with the other models being in the 0.40–0.60 range and higher as more covariates are used. These statistics are reported in the S1 Table.

## Results

**Fig 1:**
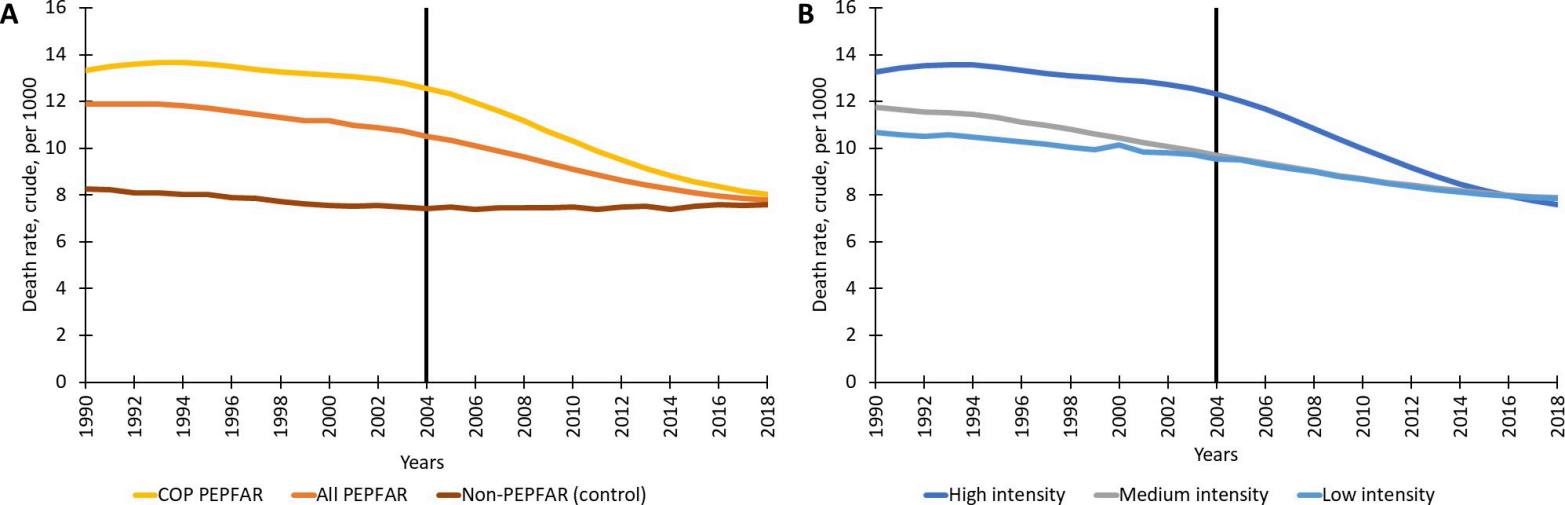
Trends in all-cause mortality rates (per 1000) for PEPFAR and control group countries. Notes: ACM = all-cause mortality; COP = country operating plans; PEPFAR = President’s Emergency Plan for AIDS Relief.

Fig 1 shows the ACM trends in the five all-PEPFAR cohorts and the control group. After the introduction of PEPFAR (2004 and after) the trends for ACM in PEPFAR country cohorts are notably different than the control. ACM rate trends from 1990–2018 indicate that both PEPFAR countries and control countries show a modest (and quite similar) decline in ACM from 1990 to the introduction of PEPFAR funding in 2004, followed by a more rapid decline in ACM for PEPFAR countries and a slight upward trend for the control group (Fig 1A). Across the three funding intensity cohorts based on cumulative PEPFAR aid per capita (high, medium, and low intensity) there is a particularly striking decline in ACM after 2004 in countries where per capita aid has been largest (high intensity), although ACM rates decrease across all groups (Fig 1B).

While the ACM trends throughout the baseline period (pre-2004) are downward sloping (lower death rates), the trends in PEPFAR cohorts continue to fall after 2004 while the control group trends become relatively flat after 2004. As required for DID validity, the down-sloping baseline trends in the control group are roughly parallel to the trends in the PEPFAR cohorts. Tests were done to confirm that the baseline trends in ACM for treatment and control groups were parallel. These test results, provided in S2 Fig materials, show that in all cohorts of the PEPFAR recipient countries, the baseline trends in PEPFAR and control countries are parallel.

Table 1 describes the characteristics of the PEPFAR cohorts and the control group. Most of the PEPFAR program funding (97%) was provided to COP countries over the 2004–2018 period. Notably, 54 of the 90 PEPFAR countries received US aid for HIV prevention prior to 2004. PEPFAR countries at baseline were poorer; spent less on health; and populations were less educated, more rural, had higher fertility rates, and had a lower life expectancy at birth than the controls. COP countries and the high PEPFAR-spending intensity countries are nearly identical (all high spending countries are also COP countries).

**Table 1:** Characteristics of PEPFAR-recipient and control countries.

	All PEPFAR funded LMICs*	COPs	Non-COP other PEPFAR	High intensity PEPFAR funding per capita group	Middle intensity PEPFAR funding per capita group	Low intensity PEPFAR funding per capita group	Control group (LMICs)**
**Number of countries**	90	31	59	30	30	30	67
**Total population (2018) in millions**	5,609.5	2,680.3	2,929.2	903.5	441.5	4,264.5	860.2
**Cumulative PEPFAR disbursements, 2004–2018 (in millions of USD)**	$40,920.2	$39,783.7	$1,136.5	$38,800.0	$1,164.6	$955.6	$8.0
**Cumulative PEPFAR disbursements per capita, 2004–2018 (in constant USD)**	$3,094.2	$2,974.1	$120.1	$3,008.0	$77.3	$8.9	$0.4
**Cumulative other donor health spending per capita (non-PEPFAR), 2004–2018 (in constant USD)**	$10,357.0	$3,948.4	$6,408.6	$4,416.8	$3,931.6	$2,008.6	$14,008.5
**Cumulative domestic health spending per capita, 2000–2016 (PPP, current international $)**	$365,733.0	$85,928.7	$279,804.3	$84,553.9	$129,107.2	$152,072.0	$588,474.8
**BL GDP per capita, PPP (constant 2011 international $)**	$5,249.3	$3,555.5	$6,154.7	$3,579.1	$5,819.4	$6,293.9	$4,654.6
**BL HIV prevalence (% of population ages 15–49)**	3.0%	7.0%	0.9%	7.4%	0.8%	0.8%	0.2%
**BL life expectancy at birth**	61.1	55.1	64.2	55.1	64.1	64.0	71.1
**BL urban population (%)**	41.7%	33.8%	45.9%	36.4%	41.0%	47.8%	58.0%
**BL school enrollment, secondary (% gross)**	52.8%	42.1%	58.2%	40.3%	59.0%	58.6%	80.4%
**BL fertility rate (births per woman)**	4.0	4.4	3.8	4.5	3.7	3.7	2.6
**Number of countries receiving US HIV aid before 2004**	54	25	29	24	16	14	2

Notes: BL = baseline (2004); COP = country operating plans; HIV = human immunodeficiency virus; LMIC = low- and middle-income countries; PEPFAR = President’s Emergency Plan for AIDS Relief; PPP = purchasing power parity; USD = US dollars.

Baseline values are the country group average for 2004. Missing data were managed by interpolation from existing data. (See S4 Text)

*Excludes 18 minimally and sporadically PEPFAR-funded countries (<$1m cumulative funding through 2018, or less than $0.50 per capita cumulative funding through 2018)

**Includes 49 LMIC countries not funded by PEPFAR and 18 minimally and sporadically PEPFAR-funded countries (<$1m cumulative funding through 2018, or less than $0.50 per capita cumulative funding through 2018).

Table 2 provides DID impact estimates for each country PEPFAR cohort on ACM using the five model specifications. Generally, the impact estimates we report from the no-covariate specification (Model 1) and the models using baseline covariates (Models 2,3) are similar in sign, significance, and order of magnitude.

**Table 2:** DID estimates of PEPFAR impact on all-cause mortality (ACM) rates in PEPFAR-recipient countries, 2004–2018.

All-Cause Mortality Rate (deaths per, 1000)	All PEPFAR	COP PEPFAR	Non-COP other PEPFAR	High program spending intensity	Medium program spending intensity	Low program spending intensity
**Mean ACM in PEPFAR countries (2004)**	10.5	12.6	9.4	12.3	9.7	9.5
**Model 1. DID model with no covariates, 1990–2018**	-2.090***	-2.883***	-1.674***	-3.081***	-1.942***	-1.247***
**Model 2. DID model with non-financial BL covariates only, 1990–2018** ^**a**^	-2.206***	-3.086***	-1.758***	-3.373***	-2.003***	-1.329***
**Model 3. DID model with non-financial and financial BL covariates, 1990–2018** ^**b**^	-2.157***	-3.036***	-1.709***	-3.324***	-1.952***	-1.281***
**Model 4. DID model with non-financial BL covariates and yearly other donor spending on health covariate, 2002–2018**	-1.814***	-2.809***	-1.302***	-2.986***	-1.324***	-1.208**
**Model 5. DID model with non-financial BL covariates and yearly other donor spending and domestic spending on health covariates, 2002–2016**	-1.072***	-1.854***	-0.629*	-2.035***	-0.638	-0.527
**Approximate % reduction in ACM** ^**c**^	10.2–21.0%	14.7–24.5%	6.7–18.7%	16.5–27.4%	6.6–20.6%	5.5–14.0%

Notes: ACM = All-cause mortality (rate of deaths per 1,000); BL = baseline (2004); COP = country operating plans; DID difference-in-difference; PEPFAR = President’s Emergency Plan for AIDS Relief.

^a^ Baseline (2004) non-financial covariates include HIV prevalence rate (% of population ages 15–49), GDP per capita (constant USD), population size, percent of urban population, secondary school enrollment (% gross), life expectancy at birth, fertility rate (births per woman), whether the country was a low-income or a middle-income country (dummy), and whether the country received HIV aid from the U.S. prior to 2004 (dummy).

^b^ Baseline (2004) financial covariates include other donor health spending and domestic health spending.

^c^ For each model the percent is calculated by dividing the coefficient by the mean of ACM in 2004 as shown in the first row of the table. The range is formed by taking the lowest and highest percentages across the five models

***p < 0.001

**p < 0.01

* p< 0.05.

Overall, we find that across all 90 PEPFAR recipient countries, the ACM rate declined by approximately 10–21% over the 2004–2018 period. The pattern of mortality effects of PEPFAR varies substantially across the cohort categories of countries. The 31 COP countries have larger reductions in ACM than the programmatic average (15–25%). We also find that PEPFAR’s impact was greater in countries with higher spending intensity. The 30 countries receiving the most aid per capita have the largest estimated PEPFAR impacts (17–27% reductions in ACM). The size of these estimated effects is very similar to the COP estimates, given the high overlap between the groups (25 countries are common to both groups). The 30 countries receiving the least PEPFAR funding have the lowest ACM rate reductions (6–14%). The middle grouping of 30 countries achieved a reduction in between the high and low groups (7–21%).

Impact estimates are higher in models using the baseline covariates, and lower for the models where yearly spending variables are included in the model (models 4 and 5).

The inclusion of the non-financial baseline covariates plus “yearly other donor spending per capita” (Model 4) shows the same general pattern as the models with only baseline covariates, with slightly smaller PEPFAR impacts. Model 5, where the yearly domestic per capita health spending covariate is added to Model 4, the results show the same general pattern of significant impact coefficients, but the impact estimates are much lower than the other models. Model 5 also has two cohorts (medium and low-spending countries) where the negative ACM coefficients were too small to be statistically significant. Full model results are presented in S1 Table.

Table 3 shows the DID model estimates of PEPFAR impact on ACM for 3 time periods: 2004–2008, 2004–2013, and 2004–2018, generally corresponding with the three different authorizing periods of the program. The same five models are estimated here, but for only two cohorts: all 90 recipient countries taken together, and COP countries. We used these estimates from the five models to calculate the average incremental impact for each time period, which is shown on the table. The incremental impact for the second 5-year period is computed by subtracting the average impact in the first period (2004–2008) from the estimated impact in the second period (2004–2013).

**Table 3:** Estimates of PEPFAR impact on all-cause mortality (deaths per 1,000), cumulative 5-year estimates.

All-Cause Mortality Rate	All PEPFAR			COP PEPFAR		
	2004–2008	2004–2013	2004–2018	2004–2008	2004–2013	2004–2018
**Model 1. DID model with no covariates, 1990–2018**	-0.945**	-1.567***	-2.090***	-0.996*	-2.016***	-2.883***
**Model 2. DID model with non-financial BL covariates only, 1990–2018** ^**a**^	-1.081***	-1.677***	-2.206***	-1.205**	-2.207***	-3.086***
**Model 3. DID model with non-financial and financial BL covariates, 1990–2018** ^**b**^	-1.027***	-1.629***	-2.157***	-1.152***	-2.157***	-3.036***
**Model 4. DID model with non-financial BL covariates and yearly other donor spending on health covariate, 2002–2018**	-0.721*	-1.294***	-1.814***	-0.961*	-1.939***	-2.809***
**Model 5. DID model with non-financial BL covariates and yearly other donor spending and domestic spending on health covariates, 2002–2016**	-0.221	-0.691*	-1.072***	-0.340	-1.215**	-1.854***
**Approximate % reduction in ACM** ^**c**^	2.1–10.3%	6.6–16.0% 10.9%	10.2–21.0%	2.7–9.6%	9.6–17.5%	14.7–24.5%
**Incremental average** ^**d**^	7.6%	5.5%	4.7%	7.4%	7.7%	6.6%

Notes: ACM = All-cause mortality (rate of deaths per 1,000); BL = baseline (2004); COP = country operating plans; DID = difference-in-difference; PEPFAR = President’s Emergency Plan for AIDS Relief.

^a^ Baseline (2004) non-financial covariates include HIV prevalence rate (% of population ages 15–49), GDP per capita (constant USD), population size, percent of urban population, secondary school enrollment (% gross), life expectancy at birth, fertility rate (births per woman), whether the country was a low-income or a middle-income country (dummy), and whether the country received HIV aid from the U.S. prior to 2004 (dummy).

^b^ Baseline (2004) financial covariates include other donor health spending and domestic health spending.

^c^ For each model the percent is calculated by dividing the coefficient by the mean of ACM in 2004 (10.5 for ALL, and 12.6 for COP). The range is formed by taking the lowest and highest percentages across the five models

^d^ The incremental impact for the first period is the average impact across the 5 models for 2004–2008. The incremental impact for the second 5-year period is computed by subtracting the average impact across models in the first period (2004–2008) from the estimated average impact across models in the second period (2004–2013). The third period incremental impact is computed by subtracting the average impact across models in the second period (2004–2013) from the estimated average impact across models in the third period (2004–2018)

***p < 0.001

**p < 0.01

* p< 0.05.

The table supports three conclusions: (1) the longer PEPFAR operates in countries, the larger the overall program impact on ACM. In each cohort the effect of PEPFAR on lives saved is larger if the program lasts 10 years, rather than just 5; and is larger if the program lasts 15 years, compared to 10; (2) for all 90 countries taken together the reduction in ACM per additional year of PEPFAR presence gets somewhat smaller per time period the longer the program operates. The highest incremental returns appear in the first 5-year phase, and progressively become somewhat smaller for the second and third phases. (3) The diminishing incremental effect sizes over time are less pronounced for COP countries than others.

For All PEPFAR countries taken together over the period 2004–2008, the average impact across all five models is an average estimated reduction in ACM by -7.6%. In the second 5-year period the average (across models) incremental impact of PEPFAR was– 5.5%. And in the third period the average incremental impact is somewhat smaller at -4.7%.

In COP countries the story from Table 3 is somewhat different. In the first 5-year period COP countries achieved an average (across models) of a -7.4% reduction in ACM–somewhat comparable to the estimate of -7.6% for all PEPFAR countries taken together. In the second period (2009–2013) the average increment across models shows a somewhat larger impact (-7.7%) in the COP countries. And, in the third period (2014–2018) the incremental ACM impact decreased to -6.6%.

In Table 3 the impact coefficients for Model 5 are insignificant for the initial period (2004–2008) for both the All Country and COP cohorts. Also, in Table 2, the Model 5 coefficients were too small to be statistically significant for the Medium and Low Spending cohorts. This model has all non -financial baseline covariates, as well as three time-varying covariates that combines three types of non-PEPFAR health spending (per capita levels of other donor health spending, domestic government health spending, and private health spending). As noted earlier, specification revealed that the time-varying covariates in both 4 and 5 were endogenous. As a consequence, these results should be viewed with caution.

## Discussion

Our results show estimated impacts of PEPFAR on the ACM rate during the program’s first 15 years of operation (2004–2018) in 90 recipient LMICs. Several programmatic decisions and other policy choices most certainly influenced these impacts. The PEPFAR program was authorized in 2003 and began operating at some scale in 2004 in some LMIC countries. Over time, the specific operational services were expanded, as was the list of recipient countries. Some countries received more aid, some less; some countries were required to participate in a rigorous annual planning and performance monitoring process (the Country Operational Plan, or COP, process), while others were not. While we cannot say what the impact of these programmatic choices were, the analyses of population and temporal segments suggest a pattern of impacts.

Our results clearly show that the COP countries, where the epidemic needs were greatest, where ACM rates were highest at baseline, and where the vast majority of funds were expended, were able to achieve the largest PEPFAR impacts on ACM. Across the 5 models the estimates of COP country impacts were reductions in ACM of 14.7–24.5%, while the other 59 non-COP countries achieved reductions in ACM of only 6.7–18.7% over the same 2004–2018 period.

The magnitude of our impact estimates on ACM rates is large and consistent with earlier research studies. Daschle & Frist [9] reported a reduction of about 33% in ACM in their assessment of 25 countries with highest HIV prevalence between 2004 and 2016. This is somewhat larger than our estimates for COP and highly funded countries. Similarly, Bendavid et al. [7] found a 10.5% annual decrease in the number of HIV deaths in 12 “focus” countries between 2004 and 2007. Although an inexact comparison, the estimated impact is only slightly higher than our estimates for the 2004–2008 period in COP countries (7.4%).

Our analysis also shows that the impact of PEPFAR presence on ACM is subject to temporal patterns. Across all the 90 recipient countries we study, ACM declined on average (the Table 3 average across the 5 models) from about 7.6% in the first 5-year period we study, to 5.5% in the second period, and 4.7% in the third 5-year period. The COP countries show similar impacts in the first 5-year period with a 7.4% reduction, followed by a slightly larger impact in the second period with a 7.7% reduction, and a smaller decline to a 6.6% reduction in the last period. This pattern of declines in PEPFAR impact in the later periods mirrors the converging patterns of ACM across the control and PEPFAR recipient countries seen in Fig 1. This pattern of slight declining impact of PEPFAR in the third period may suggest that the program enabled recipient countries to quickly begin to save and extend lives, but further increments of improvements vis á vis the control group have been more challenging to achieve in the third period. A second possibility is that this results from PEPFAR’s changes in strategy over time (e.g., shifting from an “emergency strategy” in the first five years to a “building sustainability strategy” in the second five years, and then moving to the “accountable control” of the epidemic period after 2014) [3]. Finally, the earlier years correspond to rapid increases in PEPFAR spending from 2004 to 2009 and then a plateauing after that point, which could influence the magnitude of the impact across the study periods [1].

It is important to recognize the limitations of the DID estimates we make here. While we have been able to estimate models that control for levels of other donor health sector support (model 4) and domestic health spending (model 5) we have not been able to include local country programs or changes in policy for domestic health systems that might have been taken to combat the HIV epidemic.

While we believe that the size of PEPFAR program impacts are generally consistent with other studies, it is certainly possible that our DID estimates unintentionally capture the impacts of some country specific health policies and programs that were introduced after 2003 that have also saved lives. The models do suggest that when other donor and domestic spending are controlled (model 5) the PEPFAR impact estimates are lower. Unfortunately, these donor and domestic spending variables are clearly endogenous, and their coefficients may be the results of endogeneity.

Our research examines the impacts of “PEPFAR presence” across cohorts of countries, and across time periods. More research is needed to better understand the linkages between program characteristics and lives saved. Specifically, PEPFAR intervened by providing resources to implementing partners, who in turn offered products and services to people on the ground. Country PEPFAR programs are somewhat different, and the DID estimates are not able to identify which program characteristics (other than PEPFAR presence, and which PEPFAR cohort) are driving the life-saving results. There are likely other programmatic factors affecting impact which would improve policy-maker’s understanding about what works best to save lives. Such programmatic factors may include choices about facility siting location of services for testing and treatment, the extent of country leadership support of public information campaigns about risk factors and public health measures, and other factors. Country programs are also different in terms of how budgets are allocated. Understanding more about the links between resources, services, outputs and outcomes would help to create confidence in “how PEPFAR saved lives,” and it would also help program staff understand how the overall budget allocation might be better distributed to “get more bang for the buck.” These budget variances can be used to analyze the incremental contribution of spending more on prevention, testing, care and treatment, or other areas related to broader HIV efforts.

Finally, the magnitude of the impact on ACM suggests that PEPFAR may have had positive spillover effects on ACM beyond HIV disease alone. This is plausible, given that PEPFAR has invested significantly in the recipient countries’ health systems, including the workforce and supply chains, (estimated to be more than $1 billion per year) [2]. It also seems likely that the expansion of health facility capacities, including in rural areas, may well have introduced many families to professional health services for the first time and may have contributed to ACM impacts captured in the data that are not related to HIV. Our results (not shown here) show sizable health spillover effects beyond HIV. Bendavid (2016) also mentions possible spillover effects [21]. Future research could explore this further and seek to quantify this spillover impact.

### Strengths and limitations of the study

Despite the consistency of our findings across model specifications, this study does have some limitations. The quasi-experimental design offers a convenient method for obtaining estimates with a large panel dataset, and the use of baseline and other covariates helps to adjust estimates for differences between recipient and control countries. Despite these adjustments, the control group is not ideal and the use of some minimally supported countries in the control group may make our PEPFAR impact estimates somewhat conservative in magnitude. While we control for baseline differences and yearly “other donor health spending per capita” and “domestic health spending per capita” it is still possible that the effect of PEPFAR is being systematically over- or underestimated.

Our model specifications appear reasonably stable and consistent, but there is always a risk of attributing unwarranted differences and changes in ACM to PEPFAR’s presence. We have included in models 4 and 5 time-varying covariates of non PEFAR health spending, hoping to control for potentially confounding influences of other programs and initiatives aiming to control the epidemic. The estimates of PEPFAR influence in these specifications are consistent with other models, though the PEPFAR impacts are, in some cases, somewhat smaller, particularly in Model 5. But, the Hausman test for endogeneity confirmed the problem in Models 4 and 5, making it impossible to say whether the differences in size of impact estimates in these models, particularly Model 5, are a result of endogeneity issues or something else.

Another important limitation is the inability to separate the PEPFAR effects of spending intensity from COP status. Unfortunately, there is a very high overlap between these two categories (e.g., PEPFAR demands very intense country annual planning where it provides larger sums of money).

Finally, the DID estimates presented here do not tell us about which specific features of programming are most effective in saving lives. Estimates of cohort Impacts are attributed to “PEPFAR’s presence” only. Many contributing characteristics of country-specific programming and budget allocations would provide richer data, and more refined knowledge about what is saving lives.

Despite these limitations, we believe that our estimates of PEPFAR’s impact on ACM are properly attributed to PEPFAR due to four factors: (1) the general size of ACM reductions are similar to the prior literature, particularly, Bendavid (2012) [8]; (2) the general consistency in the size of our estimates of effects of PEPFAR on ACM using different model specifications; and (3) the steps taken in our research approach to estimate patterns of PEPFAR effects across subgroups of countries with intense country planning (or not) and countries with high-, medium- and low-aid levels. These results show patterns of PEPFAR impacts we would expect to see if the PEPFAR program was working as intended. As expected, places with higher program spending have larger program effects on ACM and countries that are required to conduct rigorous annual planning and budget scrutiny (COP) have bigger impacts on ACM. (4) We also estimated all models using a double log transformation, and report results in the S1 Table materials. The estimates are quite similar and the patterns across models and cohorts are also similar to the non-log results. The log models suggest that PEPFAR has reduced ACM rates across all 90 country recipients by approximately 15–17% over the 15-year interval, somewhat higher (approximately 23–26%) in country segments where cumulative spending has been higher and/or where intensive planning has occurred, and somewhat lower (approximately 11–14%) in the other non-COP PEPFAR recipients. The log models also suggest that the life-saving impacts of the program remain substantial in the most recent, 2014–2018 period (4.9% annual reductions in all PEPFAR countries, and 7.9% in the COP countries).

## Conclusions

PEPFAR’s substantial response to the HIV/AIDS epidemic beginning in late 2003 continues to bring accessible, free, and modern tools of medicine and public health to LMIC countries fighting HIV. Using a panel data set starting in 1990, and across the program’s operations during 2004–2018 in 90 recipient countries, we estimate that PEPFAR has reduced ACM rates by 10–21% over the 15-year interval, and somewhat higher in country segments where cumulative spending has been higher and/or where intensive planning has occurred. Over the course of the first 15 years of PEPFAR operation, the life-saving impacts of the program remain very substantial in the most recent, 2014–2018 period (4.7% decrease in all PEPFAR countries over the period, and 6.6% in the COP countries). These findings suggest that continued support from PEPFAR would achieve further gains over time.

## Supporting information

S1 Text Country list by groups. (DOCX)

S2 Text Cohorts of PEPFAR countries created for analysis. (DOCX)

S3 Text Difference-in-difference (DID) methodology. (DOCX)

S4 Text Missingness. (DOCX)

S1 Table Regression results–Tables A—O. Table A in S1 Table. Summary of PEPFAR impact by country cohort from estimation of five logged models. Table B in S1 Table. Summary of adjusted R-squares of five logged and unlogged models (level) on PEPFAR impact by country cohort. Table C in S1 Table. Summary of PEPFAR impact over three periods from estimation of five logged models. Table D in S1 Table. Full model results for "All PEPFAR" group vs control: unlogged models. Table E in S1 Table. Full model results for "All PEPFAR" group vs control: logged models. Table F in S1 Table. Full model results for "COP-PEPFAR" group vs control: unlogged models. Table G in S1 Table. Full model results for "COP-PEPFAR" group vs control: logged models. Table H in S1 Table. Full model results for "Other PEPFAR" group vs control: unlogged models. Table I in S1 Table. Full model results for "Other PEPFAR" group vs control: logged models. Table J in S1 Table. Full model results for "High intensity PEPFAR" group vs control: unlogged models. Table K in S1 Table. Full model results for "High intensity PEPFAR" group vs control: logged models. Table L in S1 Table. Full model results for "Medium intensity PEPFAR" group vs control: unlogged models. Table M in S1 Table. Full model results for "Medium intensity PEPFAR" group vs control: logged models. Table N in S1 Table. Full model results for "Low intensity PEPFAR" group vs control: unlogged models. Table O in S1 Table. Full model results for “Low intensity PEPFAR” group vs control: logged models.
(DOCX)

S1 Fig Test the normal distribution of residuals derived from logged and unlogged Model 4 and Model 5—Figs A—H. Fig A in S1 Fig. Residuals from unlogged Model 4 on all PEPFAR countries. Fig B in S1 Fig. Residuals from logged Model 4 on all PEPFAR countries. Fig C in S1 Fig. Residuals from unlogged Model 4 on COP countries. Fig D in S1 Fig. Residuals from logged Model 4 on COP countries. Fig E in S1 Fig. Residuals from unlogged Model 5 on all PEPFAR countries. Fig F in S1 Fig. Residuals from logged Model 5 on all PEPFAR countries. Fig G in S1 Fig. Residuals from unlogged Model 5 on COP countries. Fig H in S1 Fig. Residuals from logged Model 5 on COP countries.
(DOCX)

S2 Fig Test the parallel assumption of ACM by country cohort. Fig A in S2 Fig. All PEPFAR countries versus control countries. Fig B in S2 Fig. COP countries versus control countries. Fig C in S2 Fig. Non-COP PEPFAR countries versus control countries. Fig D in S2 Fig. High intensity countries versus control countries. Fig E in S2 Fig. Medium intensity countries versus control countries. Fig F in S2 Fig. Low intensity countries versus control countries.
(DOCX)
