# Supplementary material for: Assessing the impact of the president’s emergency plan for AIDS relief on all-cause mortality
Source: PLOS Glob Public Health. 2024 Jan 18;4(1):e0002467. doi: 10.1371/journal.pgph.0002467 (PMC10796053; doi:10.1371/journal.pgph.0002467)
Supplement: S1 Text — (DOCX) [file pgph.0002467.s001.docx]

# S1 Text. Country list by groups

**All PEPFAR-funded LMICs**

Afghanistan

Albania

Angola

Armenia

Bangladesh

Barbados

Belize

Benin

Bolivia

Botswana

Brazil

Burkina Faso

Burundi

Cambodia

Cameroon

Central African Republic

Chad

China

Comoros

Congo, Dem. Rep.

Congo, Rep.

Cote d'Ivoire

Djibouti

Dominican Republic

El Salvador

Eritrea

Estonia

Eswatini

Ethiopia

Gabon

Gambia, The

Georgia

Ghana

Guatemala

Guinea

Guinea-Bissau

Guyana

Haiti

Honduras

India

Indonesia

Jamaica

Jordan

Kazakhstan

Kenya

Kyrgyz Republic

Lao PDR

Lesotho

Liberia

Madagascar

Malawi

Mali

Mauritania

Mexico

Moldova

Mozambique

Myanmar

Namibia

Nepal

Nicaragua

Niger

Nigeria

North Macedonia

Pakistan

Papua New Guinea

Peru

Philippines

Romania

Russian Federation

Rwanda

Samoa

Sao Tome and Principe

Senegal

Seychelles

Sierra Leone

South Africa

Suriname

Tajikistan

Tanzania

Thailand

Timor-Leste

Togo

Trinidad and Tobago

Turkmenistan

Uganda

Ukraine

Uzbekistan

Vietnam

Zambia

Zimbabwe

**COP-PEPFAR countries**

Angola

Botswana

Burundi

Cambodia

Cameroon

Congo, Dem. Rep.

Cote d'Ivoire

Dominican Republic

Eswatini

Ethiopia

Ghana

Guyana

Haiti

India

Indonesia

Kenya

Lesotho

Malawi

Mozambique

Myanmar

Namibia

Nigeria

Papua New Guinea

Rwanda

South Africa

Tanzania

Uganda

Ukraine

Vietnam

Zambia

Zimbabwe

**Non-COP PEPFAR countries**

Afghanistan

Albania

Armenia

Bangladesh

Barbados

Belize

Benin

Bolivia

Brazil

Burkina Faso

Central African Republic

Chad

China

Comoros

Congo, Rep.

Djibouti

El Salvador

Eritrea

Estonia

Gabon

Gambia, The

Georgia

Guatemala

Guinea

Guinea-Bissau

Honduras

Jamaica

Jordan

Kazakhstan

Kyrgyz Republic

Lao PDR

Liberia

Madagascar

Mali

Mauritania

Mexico

Moldova

Nepal

Nicaragua

Niger

North Macedonia

Pakistan

Peru

Philippines

Romania

Russian Federation

Samoa

Sao Tome and Principe

Senegal

Seychelles

Sierra Leone

Suriname

Tajikistan

Thailand

Timor-Leste

Togo

Trinidad and Tobago

Turkmenistan

Uzbekistan

**High-intensity PEFAR funding per capita group**

Angola

Botswana

Burundi

Cambodia

Cameroon

Cote d'Ivoire

Djibouti

Dominican Republic

Eswatini

Ethiopia

Ghana

Guyana

Haiti

Honduras

Jamaica

Kenya

Lesotho

Liberia

Malawi

Mozambique

Namibia

Nigeria

Rwanda

Senegal

South Africa

Tanzania

Uganda

Vietnam

Zambia

Zimbabwe

**Middle-intensity PEPFAR funding per capita group**

Albania

Barbados

Belize

Benin

Congo, Dem. Rep.

El Salvador

Eritrea

Estonia

Gabon

Gambia, The

Georgia

Guatemala

Guinea

Kyrgyz Republic

Lao PDR

Madagascar

Mali

Moldova

Myanmar

Nepal

Nicaragua

Papua New Guinea

Samoa

Sao Tome and Principe

Sierra Leone

Tajikistan

Thailand

Timor-Leste

Trinidad and Tobago

Ukraine

**Low-intensity PEPFAR funding per capita group**

Afghanistan

Armenia

Bangladesh

Bolivia

Brazil

Burkina Faso

Central African Republic

Chad

China

Comoros

Congo, Rep.

Guinea-Bissau

India

Indonesia

Jordan

Kazakhstan

Mauritania

Mexico

Niger

North Macedonia

Pakistan

Peru

Philippines

Romania

Russian Federation

Seychelles

Suriname

Togo

Turkmenistan

Uzbekistan

**Control group (all LMICs)**

Algeria

American Samoa

Antigua and Barbuda

Argentina

Azerbaijan

Belarus

Bhutan

Bosnia and Herzegovina

Bulgaria

Cabo Verde

Chile

Colombia

Costa Rica

Croatia

Cuba

Czech Republic

Dominica

Ecuador

Egypt, Arab Rep.

Equatorial Guinea

Fiji

Grenada

Hungary

Iran, Islamic Rep.

Iraq

Kiribati

Korea, Dem. People’s Rep.

Kosovo

Latvia

Lebanon

Libya

Lithuania

Malaysia

Maldives

Marshall Islands

Mauritius

Mayotte

Micronesia, Fed. Sts.

Mongolia

Montenegro

Morocco

Nauru

Northern Mariana Islands

Oman

Palau

Panama

Paraguay

Poland

Serbia and Montenegro

Serbia

Slovak Republic

Solomon Islands

Somalia

Sri Lanka

St. Kitts and Nevis

St. Lucia

St. Vincent and the Grenadines

Syrian Arab Republic

Tonga

Tunisia

Turkey

Tuvalu

Uruguay

Vanuatu

Venezuela, RB

West Bank and Gaza

Yemen, Rep.
