# Supplementary material for: Assessing the impact of the president’s emergency plan for AIDS relief on all-cause mortality
Source: PLOS Glob Public Health. 2024 Jan 18;4(1):e0002467. doi: 10.1371/journal.pgph.0002467 (PMC10796053; doi:10.1371/journal.pgph.0002467)
Supplement: S2 Text — (DOCX) [file pgph.0002467.s002.docx]

# S2 Text. Cohorts of PEPFAR countries created for analysis

1. **Based on intensity of PEPFAR funding.** Three equal sized groups of recipient countries (high, medium, and low) based on cumulative PEPFAR disbursements per capita over the interval 2004 to 2018.
2. **Based on OGAC programmatic planning differences.** The PEPFAR program has provided strategic budget and programmatic guidance in several ways over the years. PEPFAR’s initial group of 15 focus countries were later replaced with a group of high-burden, highly supported countries that were required to submit and negotiate annual Country Operating Plans (COP). This intense annual planning process involves country officials and OGAC staff conducting performance reviews of prior programming, goal setting, and annual budget negotiations. Since 2004, 31 of the 90 PEPFAR countries have consistently been COP recipients. These countries are studied separately from all other PEPFAR recipients.
