# Supplementary material for: Assessing the impact of the president’s emergency plan for AIDS relief on all-cause mortality
Source: PLOS Glob Public Health. 2024 Jan 18;4(1):e0002467. doi: 10.1371/journal.pgph.0002467 (PMC10796053; doi:10.1371/journal.pgph.0002467)
Supplement: S3 Text — (DOCX) [file pgph.0002467.s003.docx]

# S3 Text. Difference-in-difference (DID) methodology

The Difference in Difference method provides a direct estimate of the impacts of PEPFAR on outcomes, based on the counterfactual provided by a control group of countries. DID can be thought of as an extension of Quasi Experimental Design to account for unobserved variables potentially correlated with both an intervention and the outcomes that are assumed to remain fixed over time. This DID method has been widely used in the program evaluation literature to estimate treatment effects as a non-parametric alternative to parametric sample selection models (Wooldridge, 2002). The method can be used when pre and post data are available for countries that received PEPFAR funding and for those that did not (e.g., the control group). Characteristics of the control group countries were also measured in the baseline period. The first group of PEPFAR countries began receiving funding in late 2003, and the earliest health effects could first be measured in 2004, the beginning of our follow-up period. We also measure the outcomes for countries in the control group in the same post-2004 period. If we assume that countries may also have unobserved characteristics, λi, that are correlated with outcomes and that these characteristics remain fixed over time (e.g., unobserved health endowment), DID provides a method to control for these fixed, unobserved characteristics.

To see this, we defined the outcome equations for periods 1 and 2, as shown in equations 1a and 1b, respectively:

(1a) Y_i1_=B_0_ + B_1_X_i1_+B_2_λ_i_+є_i1_

(1b) Y_i2_=B_0_ + B_1_X_i2_+B_2_λ_i_+B_3_T_i_+є_i2_

Calculating the change in outcomes and explanatory variables between time 1 and time 2, and re-estimating the outcome equation, is equivalent to subtracting equation (1a) from (1b):

(2) (Y_i2_- Y_i1_)= (B_0_- B_0_)+ B_1_(X_i2_- X_i1_)+ B_2_ (λi- λ_i_) +B_3_T_i_+(є_i2_- є_i1_)

Which simplifies to:

(3) (Y_i2_- Y_i1_)= B_1_(X_i2_- X_i1_)+B_3_T_i_+(є_i2_- є_i1_)

In other words, the DID approach subtracts out unobserved fixed effects of countries that may be correlated with both treatment selection and outcomes.

Operationally, the DID model is easy to implement using three dummy variables in a panel data set: (1) a time dummy captures the overall differences in the mean value of the dependent variable between the baseline period (pre 2004) and the follow-up period; (2) A dummy variable for PEPFAR countries and 0 for control group countries; (3) an interaction dummy between the first two dummy variables, the coefficient on which estimates the impact of PEPFAR. We also conducted a simulation analysis of potential confounding influences of other health spending sources. Basically, we introduced two additional years covariates; (1) per capita health spending from other donors, and (2) percent of total health spending from domestic spending sources. These annual health spending data from the WHO exist only for 2002 -2018 and the DID specification is restricted to 2002 to 2018. With these additional covariates, the All PEPFAR country results showed a modest decline in the size of PEPFAR impact and a small (favorable and offsetting) impact of other donor health spending on ACM. The donor spending variable was of mixed sign and significance. The COP effect sizes were not much effected by the introduction of the spending covariates. In these simulations we are concerned about the endogeneity issue related to health spending and PEPFAR (see Farag, 2009) {^1^ In a simulation using only the 90 PEPFAR countries and years 2004-18 we estimated models of ACM and instruments for spending per capita for PEPFAR, for Other donors, and for percent Domestic spending. The instruments based on baseline data were determined to be very weak.

**References**

1. Farag M, Nandakumar A, Wallack S, et al. Does funding from donors displace government spending for health in developing countries? *Health Affairs* 2009;28(4).
