# Supplementary material for: Assessing the impact of the president’s emergency plan for AIDS relief on all-cause mortality
Source: PLOS Glob Public Health. 2024 Jan 18;4(1):e0002467. doi: 10.1371/journal.pgph.0002467 (PMC10796053; doi:10.1371/journal.pgph.0002467)
Supplement: S4 Text — (DOCX) [file pgph.0002467.s004.docx]

# S4 Text. Missingness

Missing baseline data (for the year 2004) were estimated using linear interpolation of existing data for all other years for most variables. Missing data on domestic private and government spending were calculated as a function of GDP per capita. HIV prevalence data were extracted from IHME if missing in the World Bank dataset.
