# Supplementary material for: Assessing the impact of the president’s emergency plan for AIDS relief on all-cause mortality
Source: PLOS Glob Public Health. 2024 Jan 18;4(1):e0002467. doi: 10.1371/journal.pgph.0002467 (PMC10796053; doi:10.1371/journal.pgph.0002467)
Supplement: S1 Table — Table A in S1 Table. Summary of PEPFAR impact by country cohort from estimation of five logged models. Table B in S1 Table. Summary of adjusted R-squares of five logged and unlogged models (level) on PEPFAR impact by country cohort. Table C in S1 Table. Summary of PEPFAR impact over three periods from estimation of five logged models. Table D in S1 Table. Full model results for "All PEPFAR" group vs control: unlogged models. Table E in S1 Table. Full model results for "All PEPFAR" group vs control: logged models. Table F in S1 Table. Full model results for "COP-PEPFAR" group vs control: unlogged models. Table G in S1 Table. Full model results for "COP-PEPFAR" group vs control: logged models. Table H in S1 Table. Full model results for "Other PEPFAR" group vs control: unlogged models. Table I in S1 Table. Full model results for "Other PEPFAR" group vs control: logged models. Table J in S1 Table. Full model results for "High intensity PEPFAR" group vs control: unlogged models. Table K in S1 Table. Full model results for "High intensity PEPFAR" group vs control: logged models. Table L in S1 Table. Full model results for "Medium intensity PEPFAR" group vs control: unlogged models. Table M in S1 Table. Full model results for "Medium intensity PEPFAR" group vs control: logged models. Table N in S1 Table. Full model results for "Low intensity PEPFAR" group vs control: unlogged models. Table O in S1 Table. Full model results for “Low intensity PEPFAR” group vs control: logged models. (DOCX) [file pgph.0002467.s005.docx]

# S1 Table. Regression results – Tables A - O

# Table A in S1 Table. Summary of PEPFAR impact by country cohort from estimation of five logged models

| **All-Cause Mortality Rate (deaths per, 1000)** | **All**  **PEPFAR** | **COP PEPFAR** | **Other non-COP PEPFAR** | **High program spending intensity** | **Medium program spending intensity** | **Low program spending intensity** |
| --- | --- | --- | --- | --- | --- | --- |
| **Model 1. DID model with no covariates, 1990-2018** | -0.161*** | -0.211*** | -0.135*** | -0.229*** | -0.162*** | -0.0929** |
| **Model 2. DID model with non-financial BL covariates only, 1990-2018^a^** | -0.170*** | -0.225*** | -0.141*** | -0.249*** | -0.166*** | -0.0980*** |
| **Model 3. DID model with non-financial and financial BL covariates, 1990-2018^b^** | -0.162*** | -0.218*** | -0.131*** | -0.229*** | -0.159*** | -0.0928*** |
| **Model 4. DID model with non-financial BL covariates and yearly other donor spending on health covariate, 2002-2018** | -0.172*** | -0.240*** | -0.132*** | -0.263*** | -0.129** | -0.118** |
| **Model 5. DID model with non-financial BL covariates and yearly other donor spending and domestic spending on health covariates, 2002-2016** | -0.153*** | -0.214*** | -0.116*** | -0.235*** | -0.116** | -0.102* |

Notes: ACM=All-cause mortality (rate of deaths per 1,000); BL=baseline (2004); COP=country operating plans; DID difference-in-difference; PEPFAR=President's Emergency Plan for AIDS Relief.

^a^ Baseline non-financial covariates include country income classification in 2004 (dummy), population size in 2004, GDP per capita (constant USD) in 2004, HIV prevalence rate (% of population ages 15-49) in 2004, life expectancy at birth in 2004, percent of urban population in 2004, secondary school enrollment (% gross) in 2004, fertility rate (births per woman) in 2004, and if the country received HIV aid from the U.S. prior to 2004 (dummy). Except for dummy variables, all the other covariates were in their logarithmic forms.

^b^ Financial covariates include other donor spending and domestic health spending. ***p < 0.001 **p < 0.01 * p< 0.05.

# Table B in S1 Table. Summary of adjusted R-squares of five logged and unlogged models (level) on PEPFAR impact by country cohort

| **Adjusted R-square** | **All**  **PEPFAR** | | **COP PEPFAR** | | **Other non-COP PEPFAR** | | **High program spending intensity** | | **Medium program spending intensity** | | **Low program spending intensity** | |
| --- | --- | --- | --- | --- | --- | --- | --- | --- | --- | --- | --- | --- |
|  | **Level** | **Log** | **Level** | **Log** | **Level** | **Log** | **Level** | **Log** | **Level** | **Log** | **Level** | **Log** |
| **Model 1. DID model with no covariates, 1990-2018** | 0.151 | 0.158 | 0.268 | 0.265 | 0.100 | 0.101 | 0.245 | 0.233 | 0.118 | 0.118 | 0.076 | 0.075 |
| **Model 2. DID model with non-financial BL covariates only, 1990-2018^a^** | 0.527 | 0.588 | 0.469 | 0.554 | 0.529 | 0.629 | 0.490 | 0.558 | 0.493 | 0.589 | 0.473 | 0.624 |
| **Model 3. DID model with non-financial and financial BL covariates, 1990-2018^b^** | 0.582 | 0.644 | 0.549 | 0.611 | 0.609 | 0.658 | 0.568 | 0.611 | 0.605 | 0.598 | 0.585 | 0.638 |
| **Model 4. DID model with non-financial BL covariates and yearly other donor spending on health covariate, 2002-2018** | 0.518 | 0.627 | 0.495 | 0.584 | 0.480 | 0.652 | 0.512 | 0.585 | 0.469 | 0.619 | 0.442 | 0.642 |
| **Model 5. DID model with non-financial BL covariates and yearly other donor spending and domestic spending on health covariates, 2002-2016** | 0.591 | 0.654 | 0.572 | 0.611 | 0.570 | 0.667 | 0.588 | 0.612 | 0.558 | 0.631 | 0.545 | 0.660 |

Notes: ACM=All-cause mortality (rate of deaths per 1,000); BL=baseline (2004); COP=country operating plans; DID difference-in-difference; PEPFAR=President's Emergency Plan for AIDS Relief.

^a^ Baseline non-financial covariates include country income classification in 2004 (dummy), population size in 2004, GDP per capita (constant USD) in 2004, HIV prevalence rate (% of population ages 15-49) in 2004, life expectancy at birth in 2004, percent of urban population in 2004, secondary school enrollment (% gross) in 2004, fertility rate (births per woman) in 2004, and if the country received HIV aid from the U.S. prior to 2004 (dummy).

^b^ Financial covariates include other donor spending and domestic health spending. ***p < 0.001 **p < 0.01 * p< 0.05.

# Table C in S1 Table. Summary of PEPFAR impact over three periods from estimation of five logged models

| **All-Cause Mortality Rate** | **All PEPFAR** | | | **COP PEPFAR** | | |
| --- | --- | --- | --- | --- | --- | --- |
|  | **2004-2008** | **2004-2013** | **2004-2018** | **2004-2008** | **2004-2013** | **2004-2018** |
| **Model 1. DID model with no covariates, 1990-2018** | -0.0494 | -0.108*** | -0.161*** | -0.0344 | -0.126*** | -0.211*** |
| **Model 2. DID model with non-financial BL covariates only, 1990-2018^a^** | -0.0627** | -0.117*** | -0.170*** | -0.0518 | -0.140*** | -0.225*** |
| **Model 3. DID model with non-financial and financial BL covariates, 1990-2018^b^** | -0.0538* | -0.109*** | -0.162*** | -0.0423 | -0.131*** | -0.218*** |
| **Model 4. DID model with non-financial BL covariates and yearly other donor spending on health covariate, 2002-2018** | -0.0748* | -0.122*** | -0.172*** | -0.0810 | -0.160*** | -0.240*** |
| **Model 5. DID model with non-financial BL covariates and yearly other donor spending and domestic spending on health covariates, 2002-2016** | -0.0697* | -0.119*** | -0.153*** | -0.0770 | -0.157*** | -0.214*** |

Notes: ACM=All-cause mortality (rate of deaths per 1,000); BL=baseline (2004); COP=country operating plans; DID difference-in-difference; PEPFAR=President's Emergency Plan for AIDS Relief.

^a^ Baseline non-financial covariates include country income classification in 2004 (dummy), population size in 2004, GDP per capita (constant USD) in 2004, HIV prevalence rate (% of population ages 15-49) in 2004, life expectancy at birth in 2004, percent of urban population in 2004, secondary school enrollment (% gross) in 2004, fertility rate (births per woman) in 2004, and if the country received HIV aid from the U.S. prior to 2004 (dummy).

^b^ Financial covariates include other donor spending and domestic health spending. ***p < 0.001 **p < 0.01 * p< 0.05.

# Table D in S1 Table. Full model results for "All PEPFAR" group vs control: unlogged models

| **VARIABLES** | **Model 1** | **Model 2** | **Model 3** | **Model 4** | **Model 5** |
| --- | --- | --- | --- | --- | --- |
| Time variable (=1 post-2004) | -0.369* | -0.309* | -0.358** | 0.0404 | -0.890*** |
|  | (0.181) | (0.146) | (0.139) | (0.251) | (0.251) |
| Intervention (=1 if PEPFAR) | 3.603*** | 1.336*** | 1.248*** | 2.004*** | 1.485*** |
|  | (0.167) | (0.162) | (0.154) | (0.315) | (0.302) |
| Interaction term (PEPFAR impact estimate) | -2.090*** | -2.206*** | -2.157*** | -1.814*** | -1.072*** |
|  | (0.232) | (0.184) | (0.174) | (0.316) | (0.306) |
| Country income level (=1 if middle income) |  | 0.479** | 0.685*** | 1.123*** | 1.157*** |
|  |  | (0.164) | (0.157) | (0.183) | (0.184) |
| BL Population |  | -1.40e-09*** | -1.02e-09*** | -1.08e-09** | -9.57e-10** |
|  |  | (0.000) | (0.000) | (0.000) | (0.000) |
| Other donor health spending per capita (baseline) |  |  | -0.0165*** |  |  |
|  |  |  | (0.002) |  |  |
| Other donor health spending per capita, yearly |  |  |  | -0.0164*** | -0.0104*** |
|  |  |  |  | (0.003) | (0.003) |
| Domestic health spending per capita (baseline) |  |  | 0.00716*** |  |  |
|  |  |  | (0.000) |  |  |
| Domestic health spending per capita, yearly |  |  |  |  | 0.00295*** |
|  |  |  |  |  | (0.000) |
| BL GDP per capita |  | 0.0000706*** | -0.000130*** | 0.0000304** | -0.0000579*** |
|  |  | (0.000) | (0.000) | (0.000) | (0.000) |
| BL HIV prevalence (% of population ages 15-49) |  | -0.325*** | -0.379*** | -0.246*** | -0.264*** |
|  |  | (0.016) | (0.015) | (0.018) | (0.018) |
| BL life expectancy at birth |  | -0.592*** | -0.632*** | -0.569*** | -0.595*** |
|  |  | (0.013) | (0.013) | (0.015) | (0.015) |
| BL Urban population (%) |  | -0.00150 | -0.0227*** | 0.000974 | -0.00968** |
|  |  | (0.003) | (0.003) | (0.004) | (0.004) |
| BL School enrollment, secondary (% gross) |  | 0.00665* | 0.00561 | 0.0158*** | 0.0137*** |
|  |  | (0.003) | (0.003) | (0.004) | (0.004) |
| BL Fertility rate (births per woman) |  | -1.171*** | -1.015*** | -1.431*** | -1.251*** |
|  |  | (0.063) | (0.060) | (0.070) | (0.071) |
| Recipient of US HIV aid before 2004 (=1 if yes) |  | -0.137 | 0.0629 | -0.772*** | -0.561*** |
|  |  | (0.113) | (0.108) | (0.126) | (0.127) |
| Constant | 7.848*** | 51.38*** | 53.68*** | 49.21*** | 50.90*** |
|  | (0.130) | (1.059) | (1.005) | (1.192) | (1.195) |
| Observations | 4284 | 3961 | 3932 | 2318 | 2025 |
| Adjusted R-squared | 0.151 | 0.527 | 0.582 | 0.518 | 0.591 |

Notes: Standard errors in parentheses; ***p < 0.001   **p < 0.01 * p< 0.05. Source: Our data came from four publicly available datasets: World Bank’s World Development Indicators; U.S. government’s foreignassistance.gov database; OECD Creditor Reporting System database; and the Institute of Health Metrics and Evaluation GBD Result’s Tool.

# Table E in S1 Table. Full model results for "All PEPFAR" group vs control: logged models

| **VARIABLES** | **Model 1** | **Model 2** | **Model 3** | **Model 4** | **Model 5** |
| --- | --- | --- | --- | --- | --- |
| Time variable (=1 post-2004) | -0.0592** | -0.0549*** | -0.0747*** | 0.0325 | 0.0511* |
|  | (0.018) | (0.014) | (0.014) | (0.025) | (0.025) |
| Intervention (=1 if PEPFAR) | 0.365*** | 0.106*** | 0.133*** | 0.214*** | 0.204*** |
|  | (0.017) | (0.015) | (0.015) | (0.031) | (0.031) |
| Interaction term (PEPFAR impact estimate) | -0.161*** | -0.170*** | -0.162*** | -0.172*** | -0.153*** |
|  | (0.024) | (0.017) | (0.017) | (0.031) | (0.031) |
| Country income level (=1 if middle income) |  | -0.0221 | -0.00543 | 0.0789*** | 0.0993*** |
|  |  | (0.016) | (0.015) | (0.019) | (0.020) |
| ln(BL Population) |  | -0.0218*** | -0.0219*** | -0.0437*** | -0.0417*** |
|  |  | (0.002) | (0.003) | (0.003) | (0.003) |
| ln(other donor health spending per capita, baseline) |  |  | -0.00315 |  |  |
|  |  |  | (0.004) |  |  |
| ln(other donor health spending per capita, yearly) |  |  |  | -0.0511*** | -0.0511*** |
|  |  |  |  | (0.004) | (0.004) |
| ln(domestic health spending per capita, baseline) |  |  | 0.00301 |  |  |
|  |  |  | (0.011) |  |  |
| ln(domestic health spending per capita, yearly) |  |  |  |  | -0.0537*** |
|  |  |  |  |  | (0.012) |
| ln(BL GDP per capita) |  | -0.0449*** | -0.0527*** | -0.116*** | -0.0737*** |
|  |  | (0.009) | (0.012) | (0.012) | (0.015) |
| ln(BL HIV prevalence, % of population ages 15-49) |  | -0.00117 | 0.00184 | -0.000209 | 0.0000152 |
|  |  | (0.002) | (0.002) | (0.002) | (0.002) |
| ln(BL life expectancy at birth) |  | -2.303*** | -1.963*** | -2.499*** | -2.556*** |
|  |  | (0.051) | (0.050) | (0.060) | (0.062) |
| ln(BL Urban population, %) |  | 0.0721*** | 0.0335** | 0.0775*** | 0.0817*** |
|  |  | (0.013) | (0.012) | (0.015) | (0.015) |
| ln(BL School enrollment, secondary, % gross) |  | -0.231*** | -0.145*** | -0.141*** | -0.144*** |
|  |  | (0.014) | (0.014) | (0.017) | (0.018) |
| ln(BL Fertility rate, births per woman) |  | -0.613*** | -0.323*** | -0.610*** | -0.623*** |
|  |  | (0.016) | (0.018) | (0.020) | (0.022) |
| Recipient of US HIV aid before 2004 (=1 if yes) |  | 0.0137 | 0.00683 | -0.00712 | -0.0124 |
|  |  | (0.011) | (0.011) | (0.014) | (0.014) |
| Constant | 1.992*** | 13.81*** | 11.87*** | 15.03*** | 15.16*** |
|  | (0.013) | (0.219) | (0.225) | (0.259) | (0.268) |
| Observations | 4284 | 3961 | 3548 | 2318 | 2025 |
| Adjusted R-squared | 0.158 | 0.588 | 0.644 | 0.627 | 0.654 |

Notes: Standard errors in parentheses; ***p < 0.001   **p < 0.01 * p< 0.05. Source: Our data came from four publicly available datasets: World Bank’s World Development Indicators; U.S. government’s foreignassistance.gov database; OECD Creditor Reporting System database; and the Institute of Health Metrics and Evaluation GBD Result’s Tool.

# Table F in S1 Table. Full model results for "COP-PEPFAR" group vs control: unlogged models

| **VARIABLES** | **Model 1** | **Model 2** | **Model 3** | **Model 4** | **Model 5** |
| --- | --- | --- | --- | --- | --- |
| Time variable (=1 post-2004) | -0.369* | -0.310 | -0.360* | 0.0158 | -0.899*** |
|  | (0.172) | (0.158) | (0.148) | (0.269) | (0.272) |
| Intervention (=1 if PEPFAR) | 5.480*** | 2.260*** | 1.966*** | 3.578*** | 2.679*** |
|  | (0.210) | (0.338) | (0.315) | (0.519) | (0.505) |
| Interaction term (PEPFAR impact estimate) | -2.883*** | -3.086*** | -3.036*** | -2.809*** | -1.854*** |
|  | (0.292) | (0.262) | (0.244) | (0.444) | (0.429) |
| Country income level (=1 if middle income) |  | -0.450 | 0.200 | 0.156 | 0.384 |
|  |  | (0.243) | (0.229) | (0.268) | (0.270) |
| BL Population |  | -2.76e-09*** | -1.81e-09*** | -1.75e-09** | -1.25e-09* |
|  |  | (0.000) | (0.000) | (0.000) | (0.000) |
| Other donor health spending per capita (baseline) |  |  | -0.0139*** |  |  |
|  |  |  | (0.003) |  |  |
| Other donor health spending per capita, yearly |  |  |  | -0.0110** | -0.00694 |
|  |  |  |  | (0.004) | (0.004) |
| Domestic health spending per capita (baseline) |  |  | 0.00849*** |  |  |
|  |  |  | (0.000) |  |  |
| Domestic health spending per capita, yearly |  |  |  |  | 0.00294*** |
|  |  |  |  |  | (0.000) |
| BL GDP per capita |  | 0.0000576*** | -0.000172*** | 0.0000114 | -0.0000687*** |
|  |  | (0.000) | (0.000) | (0.000) | (0.000) |
| BL HIV prevalence (% of population ages 15-49) |  | -0.288*** | -0.346*** | -0.220*** | -0.241*** |
|  |  | (0.021) | (0.020) | (0.023) | (0.024) |
| BL life expectancy at birth |  | -0.545*** | -0.594*** | -0.545*** | -0.578*** |
|  |  | (0.022) | (0.021) | (0.024) | (0.024) |
| BL Urban population (%) |  | 0.0117** | -0.0167*** | 0.0160*** | 0.00214 |
|  |  | (0.004) | (0.004) | (0.005) | (0.005) |
| BL School enrollment, secondary (% gross) |  | 0.0165*** | 0.0116* | 0.0339*** | 0.0315*** |
|  |  | (0.005) | (0.005) | (0.005) | (0.005) |
| BL Fertility rate (births per woman) |  | -1.165*** | -0.851*** | -1.382*** | -1.127*** |
|  |  | (0.098) | (0.092) | (0.108) | (0.109) |
| Recipient of US HIV aid before 2004 (=1 if yes) |  | 0.0193 | 0.0290 | -1.240*** | -1.086*** |
|  |  | (0.229) | (0.212) | (0.250) | (0.251) |
| Constant | 7.848*** | 47.46*** | 50.01*** | 46.17*** | 48.13*** |
|  | (0.124) | (1.761) | (1.642) | (1.944) | (1.954) |
| Observations | 2573 | 2308 | 2279 | 1349 | 1170 |
| Adjusted R-squared | 0.268 | 0.469 | 0.549 | 0.495 | 0.572 |

Notes: Standard errors in parentheses; ***p < 0.001   **p < 0.01 * p< 0.05. Source: Our data came from four publicly available datasets: World Bank’s World Development Indicators; U.S. government’s foreignassistance.gov database; OECD Creditor Reporting System database; and the Institute of Health Metrics and Evaluation GBD Result’s Tool.

# Table G in S1 Table. Full model results for "COP-PEPFAR" group vs control: logged models

| **VARIABLES** | **Model 1** | **Model 2** | **Model 3** | **Model 4** | **Model 5** |
| --- | --- | --- | --- | --- | --- |
| Time variable (=1 post-2004) | -0.0592*** | -0.0560*** | -0.0753*** | 0.0247 | 0.0425 |
|  | (0.018) | (0.015) | (0.015) | (0.028) | (0.029) |
| Intervention (=1 if PEPFAR) | 0.535*** | 0.0562 | 0.121*** | 0.250*** | 0.222*** |
|  | (0.022) | (0.033) | (0.032) | (0.054) | (0.055) |
| Interaction term (PEPFAR impact estimate) | -0.211*** | -0.225*** | -0.218*** | -0.240*** | -0.214*** |
|  | (0.030) | (0.024) | (0.024) | (0.046) | (0.045) |
| Country income level (=1 if middle income) |  | 0.0216 | -0.00373 | 0.0971*** | 0.114*** |
|  |  | (0.024) | (0.023) | (0.029) | (0.030) |
| ln(BL Population) |  | -0.00953** | -0.00160 | -0.0290*** | -0.0258*** |
|  |  | (0.004) | (0.005) | (0.005) | (0.005) |
| ln(other donor health spending per capita, baseline) |  |  | 0.0167** |  |  |
|  |  |  | (0.005) |  |  |
| ln(other donor health spending per capita, yearly) |  |  |  | -0.0426*** | -0.0431*** |
|  |  |  |  | (0.005) | (0.005) |
| ln(domestic health spending per capita, baseline) |  |  | 0.0258 |  |  |
|  |  |  | (0.018) |  |  |
| ln(domestic health spending per capita, yearly) |  |  |  |  | -0.0483* |
|  |  |  |  |  | (0.019) |
| ln(BL GDP per capita) |  | -0.0903*** | -0.0688*** | -0.161*** | -0.115*** |
|  |  | (0.013) | (0.020) | (0.017) | (0.023) |
| ln(BL HIV prevalence, % of population ages 15-49) |  | 0.000955 | 0.00679** | 0.00352 | 0.00354 |
|  |  | (0.003) | (0.003) | (0.003) | (0.003) |
| ln(BL life expectancy at birth) |  | -2.264*** | -1.723*** | -2.489*** | -2.567*** |
|  |  | (0.077) | (0.078) | (0.094) | (0.097) |
| ln(BL Urban population, %) |  | 0.0701*** | 0.0340 | 0.0984*** | 0.0922*** |
|  |  | (0.019) | (0.018) | (0.023) | (0.024) |
| ln(BL School enrollment, secondary, % gross) |  | -0.236*** | -0.0989*** | -0.0797** | -0.0863** |
|  |  | (0.022) | (0.021) | (0.026) | (0.028) |
| ln(BL Fertility rate, births per woman) |  | -0.696*** | -0.240*** | -0.645*** | -0.655*** |
|  |  | (0.024) | (0.030) | (0.031) | (0.034) |
| Recipient of US HIV aid before 2004 (=1 if yes) |  | 0.0365 | -0.00601 | -0.0548* | -0.0512 |
|  |  | (0.022) | (0.021) | (0.027) | (0.028) |
| Constant | 1.992*** | 13.95*** | 10.30*** | 14.87*** | 15.06*** |
|  | (0.013) | (0.351) | (0.375) | (0.429) | (0.446) |
| Observations | 2573 | 2308 | 1982 | 1349 | 1170 |
| Adjusted R-squared | 0.265 | 0.554 | 0.611 | 0.584 | 0.611 |

Notes: Standard errors in parentheses; ***p < 0.001   **p < 0.01 * p< 0.05. Source: Our data came from four publicly available datasets: World Bank’s World Development Indicators; U.S. government’s foreignassistance.gov database; OECD Creditor Reporting System database; and the Institute of Health Metrics and Evaluation GBD Result’s Tool.

# Table H in S1 Table. Full model results for "Other PEPFAR" group vs control: unlogged models

| **VARIABLES** | **Model 1** | **Model 2** | **Model 3** | **Model 4** | **Model 5** |
| --- | --- | --- | --- | --- | --- |
| Time variable (=1 post-2004) | -0.369* | -0.309* | -0.358** | 0.0275 | -0.973*** |
|  | (0.167) | (0.130) | (0.120) | (0.243) | (0.239) |
| Intervention (=1 if PEPFAR) | 2.617*** | 1.428*** | 1.144*** | 1.735*** | 1.135*** |
|  | (0.169) | (0.156) | (0.144) | (0.331) | (0.312) |
| Interaction term (PEPFAR impact estimate) | -1.674*** | -1.758*** | -1.709*** | -1.302*** | -0.629* |
|  | (0.236) | (0.178) | (0.164) | (0.333) | (0.315) |
| Country income level (=1 if middle income) |  | 0.721*** | 0.905*** | 1.487*** | 1.423*** |
|  |  | (0.176) | (0.165) | (0.215) | (0.213) |
| BL Population |  | -1.28e-09*** | -6.24e-10 | -1.32e-09** | -1.11e-09* |
|  |  | (0.000) | (0.000) | (0.000) | (0.000) |
| Other donor health spending per capita (baseline) |  |  | -0.0173*** |  |  |
|  |  |  | (0.002) |  |  |
| Other donor health spending per capita, yearly |  |  |  | -0.0138*** | -0.00792** |
|  |  |  |  | (0.003) | (0.003) |
| Domestic health spending per capita (baseline) |  |  | 0.00716*** |  |  |
|  |  |  | (0.000) |  |  |
| Domestic health spending per capita, yearly |  |  |  |  | 0.00317*** |
|  |  |  |  |  | (0.000) |
| BL GDP per capita |  | 0.0000662*** | -0.000129*** | 0.0000350** | -0.0000588*** |
|  |  | (0.000) | (0.000) | (0.000) | (0.000) |
| BL HIV prevalence (% of population ages 15-49) |  | -0.360*** | -0.334*** | -0.279*** | -0.174* |
|  |  | (0.061) | (0.057) | (0.074) | (0.074) |
| BL life expectancy at birth |  | -0.610*** | -0.657*** | -0.581*** | -0.601*** |
|  |  | (0.015) | (0.013) | (0.018) | (0.017) |
| BL Urban population (%) |  | -0.00423 | -0.0277*** | -0.00539 | -0.0190*** |
|  |  | (0.003) | (0.003) | (0.004) | (0.004) |
| BL School enrollment, secondary (% gross) |  | 0.00560 | 0.00886** | 0.0106* | 0.0107** |
|  |  | (0.003) | (0.003) | (0.004) | (0.004) |
| BL Fertility rate (births per woman) |  | -1.380*** | -1.126*** | -1.593*** | -1.342*** |
|  |  | (0.063) | (0.058) | (0.077) | (0.077) |
| Recipient of US HIV aid before 2004 (=1 if yes) |  | -0.469*** | -0.0415 | -0.725*** | -0.336* |
|  |  | (0.121) | (0.113) | (0.146) | (0.146) |
| Constant | 7.848*** | 53.29*** | 55.57*** | 50.85*** | 51.94*** |
|  | (0.120) | (1.123) | (1.034) | (1.372) | (1.348) |
| Observations | 3385 | 3120 | 3091 | 1825 | 1588 |
| Adjusted R-squared | 0.100 | 0.529 | 0.609 | 0.480 | 0.570 |

Notes: Standard errors in parentheses; ***p < 0.001   **p < 0.01 * p< 0.05. Source: Our data came from four publicly available datasets: World Bank’s World Development Indicators; U.S. government’s foreignassistance.gov database; OECD Creditor Reporting System database; and the Institute of Health Metrics and Evaluation GBD Result’s Tool.

# Table I in S1 Table. Full model results for "Other PEPFAR" group vs control: logged models

| **VARIABLES** | **Model 1** | **Model 2** | **Model 3** | **Model 4** | **Model 5** |
| --- | --- | --- | --- | --- | --- |
| Time variable (=1 post-2004) | -0.0592** | -0.0546*** | -0.0760*** | 0.0210 | 0.0201 |
|  | (0.018) | (0.012) | (0.013) | (0.023) | (0.025) |
| Intervention (=1 if PEPFAR) | 0.275*** | 0.0888*** | 0.100*** | 0.156*** | 0.149*** |
|  | (0.018) | (0.015) | (0.015) | (0.032) | (0.032) |
| Interaction term (PEPFAR impact estimate) | -0.135*** | -0.141*** | -0.131*** | -0.132*** | -0.116*** |
|  | (0.026) | (0.017) | (0.017) | (0.032) | (0.032) |
| Country income level (=1 if middle income) |  | 0.0302 | 0.0130 | 0.144*** | 0.141*** |
|  |  | (0.017) | (0.017) | (0.020) | (0.022) |
| ln(BL Population) |  | -0.0209*** | -0.00896** | -0.0417*** | -0.0401*** |
|  |  | (0.002) | (0.003) | (0.003) | (0.004) |
| ln(other donor health spending per capita, baseline) |  |  | 0.0180*** |  |  |
|  |  |  | (0.004) |  |  |
| ln(other donor health spending per capita, yearly) |  |  |  | -0.0378*** | -0.0373*** |
|  |  |  |  | (0.004) | (0.004) |
| ln(domestic health spending per capita, baseline) |  |  | 0.0648*** |  |  |
|  |  |  | (0.012) |  |  |
| ln(domestic health spending per capita, yearly) |  |  |  |  | -0.00491 |
|  |  |  |  |  | (0.014) |
| ln(BL GDP per capita) |  | 0.00338 | -0.0333** | -0.0748*** | -0.0651*** |
|  |  | (0.009) | (0.012) | (0.013) | (0.015) |
| ln(BL HIV prevalence, % of population ages 15-49) |  | 0.00823*** | 0.0106*** | 0.00892*** | 0.00833*** |
|  |  | (0.002) | (0.002) | (0.002) | (0.002) |
| ln(BL life expectancy at birth) |  | -3.427*** | -3.001*** | -3.300*** | -3.371*** |
|  |  | (0.071) | (0.072) | (0.087) | (0.093) |
| ln(BL Urban population, %) |  | -0.0610*** | -0.110*** | -0.0334 | -0.0360 |
|  |  | (0.014) | (0.015) | (0.018) | (0.019) |
| ln(BL School enrollment, secondary, % gross) |  | -0.110*** | -0.0400* | -0.0968*** | -0.0978*** |
|  |  | (0.016) | (0.016) | (0.020) | (0.021) |
| ln(BL Fertility rate, births per woman) |  | -0.698*** | -0.437*** | -0.716*** | -0.707*** |
|  |  | (0.016) | (0.018) | (0.021) | (0.023) |
| Recipient of US HIV aid before 2004 (=1 if yes) |  | 0.0260* | 0.0200 | 0.0214 | 0.0232 |
|  |  | (0.012) | (0.012) | (0.015) | (0.016) |
| Constant | 1.992*** | 18.20*** | 15.79*** | 18.35*** | 18.58*** |
|  | (0.013) | (0.286) | (0.292) | (0.347) | (0.371) |
| Observations | 3385 | 3120 | 2736 | 1825 | 1588 |
| Adjusted R-squared | 0.101 | 0.629 | 0.658 | 0.652 | 0.667 |

Notes: Standard errors in parentheses; ***p < 0.001   **p < 0.01 * p< 0.05. Source: Our data came from four publicly available datasets: World Bank’s World Development Indicators; U.S. government’s foreignassistance.gov database; OECD Creditor Reporting System database; and the Institute of Health Metrics and Evaluation GBD Result’s Tool.

# Table J in S1 Table. Full model results for "High intensity PEPFAR" group vs control: unlogged models

| **VARIABLES** | **Model 1** | **Model 2** | **Model 3** | **Model 4** | **Model 5** |
| --- | --- | --- | --- | --- | --- |
| Time variable (=1 post-2004) | -0.369* | -0.310* | -0.358* | 0.0185 | -0.870** |
|  | (0.175) | (0.156) | (0.146) | (0.262) | (0.265) |
| Intervention (=1 if PEPFAR) | 5.334*** | 3.122*** | 2.427*** | 4.257*** | 3.165*** |
|  | (0.215) | (0.344) | (0.322) | (0.520) | (0.508) |
| Interaction term (PEPFAR impact estimate) | -3.081*** | -3.373*** | -3.324*** | -2.986*** | -2.035*** |
|  | (0.300) | (0.265) | (0.246) | (0.443) | (0.427) |
| Country income level (=1 if middle income) |  | -0.363 | 0.0299 | 0.339 | 0.431 |
|  |  | (0.248) | (0.231) | (0.268) | (0.270) |
| BL Population |  | -9.45e-09** | -1.32e-08*** | -4.69e-09 | -5.69e-09 |
|  |  | (0.000) | (0.000) | (0.000) | (0.000) |
| Other donor health spending per capita (baseline) |  |  | -0.0151*** |  |  |
|  |  |  | (0.003) |  |  |
| Other donor health spending per capita, yearly |  |  |  | -0.0115** | -0.00786* |
|  |  |  |  | (0.004) | (0.004) |
| Domestic health spending per capita (baseline) |  |  | 0.00851*** |  |  |
|  |  |  | (0.000) |  |  |
| Domestic health spending per capita, yearly |  |  |  |  | 0.00286*** |
|  |  |  |  |  | (0.000) |
| BL GDP per capita |  | 0.0000642*** | -0.000166*** | 0.0000195 | -0.0000578*** |
|  |  | (0.000) | (0.000) | (0.000) | (0.000) |
| BL HIV prevalence (% of population ages 15-49) |  | -0.312*** | -0.343*** | -0.222*** | -0.222*** |
|  |  | (0.022) | (0.021) | (0.024) | (0.024) |
| BL life expectancy at birth |  | -0.549*** | -0.576*** | -0.530*** | -0.546*** |
|  |  | (0.021) | (0.019) | (0.022) | (0.022) |
| BL Urban population (%) |  | 0.00558 | -0.0206*** | 0.00775 | -0.00459 |
|  |  | (0.004) | (0.004) | (0.005) | (0.005) |
| BL School enrollment, secondary (% gross) |  | 0.00542 | 0.00548 | 0.0195*** | 0.0199*** |
|  |  | (0.005) | (0.005) | (0.005) | (0.005) |
| BL Fertility rate (births per woman) |  | -1.377*** | -0.860*** | -1.599*** | -1.223*** |
|  |  | (0.103) | (0.099) | (0.112) | (0.116) |
| Recipient of US HIV aid before 2004 (=1 if yes) |  | -0.682** | -0.376 | -1.699*** | -1.408*** |
|  |  | (0.250) | (0.232) | (0.270) | (0.271) |
| Constant | 7.848*** | 49.49*** | 49.68*** | 47.05*** | 47.32*** |
|  | (0.126) | (1.673) | (1.555) | (1.823) | (1.825) |
| Observations | 2544 | 2250 | 2221 | 1315 | 1140 |
| Adjusted R-squared | 0.245 | 0.490 | 0.568 | 0.512 | 0.588 |

Notes: Standard errors in parentheses; ***p < 0.001   **p < 0.01 * p< 0.05. Source: Our data came from four publicly available datasets: World Bank’s World Development Indicators; U.S. government’s foreignassistance.gov database; OECD Creditor Reporting System database; and the Institute of Health Metrics and Evaluation GBD Result’s Tool.

# Table K in S1 Table. Full model results for "High intensity PEPFAR" group vs control: logged models

| **VARIABLES** | **Model 1** | **Model 2** | **Model 3** | **Model 4** | **Model 5** |
| --- | --- | --- | --- | --- | --- |
| Time variable (=1 post-2004) | -0.0592** | -0.0558*** | -0.0754*** | 0.0250 | 0.0413 |
|  | (0.018) | (0.015) | (0.015) | (0.028) | (0.029) |
| Intervention (=1 if PEPFAR) | 0.513*** | 0.0952** | 0.105** | 0.238*** | 0.233*** |
|  | (0.023) | (0.034) | (0.033) | (0.056) | (0.057) |
| Interaction term (PEPFAR impact estimate) | -0.229*** | -0.249*** | -0.229*** | -0.263*** | -0.235*** |
|  | (0.031) | (0.025) | (0.024) | (0.047) | (0.046) |
| Country income level (=1 if middle income) |  | 0.000130 | -0.0173 | 0.0792** | 0.0962** |
|  |  | (0.024) | (0.023) | (0.029) | (0.031) |
| ln(BL Population) |  | -0.0125*** | -0.00507 | -0.0345*** | -0.0301*** |
|  |  | (0.004) | (0.005) | (0.005) | (0.005) |
| ln(other donor health spending per capita, baseline) |  |  | 0.0182** |  |  |
|  |  |  | (0.006) |  |  |
| ln(other donor health spending per capita, yearly) |  |  |  | -0.0428*** | -0.0434*** |
|  |  |  |  | (0.005) | (0.005) |
| ln(domestic health spending per capita, baseline) |  |  | 0.0494* |  |  |
|  |  |  | (0.020) |  |  |
| ln(domestic health spending per capita, yearly) |  |  |  |  | -0.0445* |
|  |  |  |  |  | (0.021) |
| ln(BL GDP per capita) |  | -0.0725*** | -0.0718*** | -0.136*** | -0.0968*** |
|  |  | (0.014) | (0.022) | (0.018) | (0.024) |
| ln(BL HIV prevalence, % of population ages 15-49) |  | 0.00265 | 0.0100*** | 0.00633 | 0.00557 |
|  |  | (0.003) | (0.003) | (0.003) | (0.004) |
| ln(BL life expectancy at birth) |  | -2.228*** | -1.778*** | -2.483*** | -2.532*** |
|  |  | (0.072) | (0.071) | (0.087) | (0.090) |
| ln(BL Urban population, %) |  | 0.0665*** | 0.0198 | 0.0906*** | 0.0863*** |
|  |  | (0.018) | (0.018) | (0.022) | (0.023) |
| ln(BL School enrollment, secondary, % gross) |  | -0.242*** | -0.112*** | -0.0982*** | -0.102*** |
|  |  | (0.022) | (0.021) | (0.026) | (0.028) |
| ln(BL Fertility rate, births per woman) |  | -0.678*** | -0.253*** | -0.635*** | -0.640*** |
|  |  | (0.024) | (0.031) | (0.032) | (0.036) |
| Recipient of US HIV aid before 2004 (=1 if yes) |  | -0.0134 | -0.0245 | -0.0469 | -0.0563 |
|  |  | (0.023) | (0.023) | (0.028) | (0.030) |
| Constant | 1.992*** | 13.73*** | 10.62*** | 14.83*** | 14.88*** |
|  | (0.013) | (0.335) | (0.361) | (0.410) | (0.430) |
| Observations | 2544 | 2250 | 1953 | 1315 | 1140 |
| Adjusted R-squared | 0.233 | 0.558 | 0.611 | 0.585 | 0.612 |

Notes: Standard errors in parentheses; ***p < 0.001   **p < 0.01 * p< 0.05. Source: Our data came from four publicly available datasets: World Bank’s World Development Indicators; U.S. government’s foreignassistance.gov database; OECD Creditor Reporting System database; and the Institute of Health Metrics and Evaluation GBD Result’s Tool.

# Table L in S1 Table. Full model results for "Medium intensity PEPFAR" group vs control: unlogged models

| **VARIABLES** | **Model 1** | **Model 2** | **Model 3** | **Model 4** | **Model 5** |
| --- | --- | --- | --- | --- | --- |
| Time variable (=1 post-2004) | -0.369* | -0.308* | -0.358** | 0.0181 | -0.972*** |
|  | (0.162) | (0.130) | (0.117) | (0.244) | (0.241) |
| Intervention (=1 if PEPFAR) | 3.113*** | 1.574*** | 1.379*** | 1.712*** | 1.071** |
|  | (0.199) | (0.192) | (0.171) | (0.400) | (0.375) |
| Interaction term (PEPFAR impact estimate) | -1.942*** | -2.003*** | -1.952*** | -1.324*** | -0.638 |
|  | (0.277) | (0.213) | (0.190) | (0.399) | (0.375) |
| Country income level (=1 if middle income) |  | 0.0859 | 0.365 | 0.848** | 0.825** |
|  |  | (0.210) | (0.191) | (0.258) | (0.254) |
| BL Population |  | -1.42e-08*** | -8.95e-09** | -1.78e-08*** | -1.37e-08*** |
|  |  | (0.000) | (0.000) | (0.000) | (0.000) |
| Other donor health spending per capita (baseline) |  |  | -0.0160*** |  |  |
|  |  |  | (0.002) |  |  |
| Other donor health spending per capita, yearly |  |  |  | -0.0117*** | -0.00644* |
|  |  |  |  | (0.003) | (0.003) |
| Domestic health spending per capita (baseline) |  |  | 0.00826*** |  |  |
|  |  |  | (0.000) |  |  |
| Domestic health spending per capita, yearly |  |  |  |  | 0.00317*** |
|  |  |  |  |  | (0.000) |
| BL GDP per capita |  | 0.0000295** | -0.000202*** | -0.00000806 | -0.000105*** |
|  |  | (0.000) | (0.000) | (0.000) | (0.000) |
| BL HIV prevalence (% of population ages 15-49) |  | -0.0179 | 0.162 | 0.0608 | 0.256* |
|  |  | (0.104) | (0.094) | (0.126) | (0.125) |
| BL life expectancy at birth |  | -0.581*** | -0.626*** | -0.545*** | -0.570*** |
|  |  | (0.017) | (0.016) | (0.021) | (0.021) |
| BL Urban population (%) |  | 0.0148*** | -0.0124*** | 0.0148*** | 0.000168 |
|  |  | (0.003) | (0.003) | (0.004) | (0.004) |
| BL School enrollment, secondary (% gross) |  | 0.0361*** | 0.0379*** | 0.0434*** | 0.0437*** |
|  |  | (0.004) | (0.003) | (0.005) | (0.005) |
| BL Fertility rate (births per woman) |  | -1.262*** | -0.869*** | -1.420*** | -1.147*** |
|  |  | (0.071) | (0.065) | (0.087) | (0.087) |
| Recipient of US HIV aid before 2004 (=1 if yes) |  | 0.226 | 0.175 | 0.0179 | 0.189 |
|  |  | (0.168) | (0.149) | (0.203) | (0.200) |
| Constant | 7.848*** | 48.46*** | 50.26*** | 45.26*** | 46.69*** |
|  | (0.117) | (1.326) | (1.188) | (1.621) | (1.589) |
| Observations | 2544 | 2337 | 2308 | 1366 | 1183 |
| Adjusted R-squared | 0.118 | 0.493 | 0.605 | 0.469 | 0.558 |

Notes: Standard errors in parentheses; ***p < 0.001   **p < 0.01 * p< 0.05. Source: Our data came from four publicly available datasets: World Bank’s World Development Indicators; U.S. government’s foreignassistance.gov database; OECD Creditor Reporting System database; and the Institute of Health Metrics and Evaluation GBD Result’s Tool.

# Table M in S1 Table. Full model results for "Medium intensity PEPFAR" group vs control: logged models

| **VARIABLES** | **Model 1** | **Model 2** | **Model 3** | **Model 4** | **Model 5** |
| --- | --- | --- | --- | --- | --- |
| Time variable (=1 post-2004) | -0.0592** | -0.0547*** | -0.0758*** | 0.0178 | 0.0328 |
|  | (0.018) | (0.013) | (0.013) | (0.025) | (0.026) |
| Intervention (=1 if PEPFAR) | 0.331*** | 0.109*** | 0.136*** | 0.150*** | 0.146*** |
|  | (0.022) | (0.019) | (0.018) | (0.040) | (0.040) |
| Interaction term (PEPFAR impact estimate) | -0.162*** | -0.166*** | -0.159*** | -0.129** | -0.116** |
|  | (0.031) | (0.021) | (0.021) | (0.040) | (0.040) |
| Country income level (=1 if middle income) |  | 0.0379 | 0.0149 | 0.168*** | 0.173*** |
|  |  | (0.021) | (0.020) | (0.025) | (0.027) |
| ln(BL Population) |  | -0.0184*** | -0.00132 | -0.0357*** | -0.0331*** |
|  |  | (0.003) | (0.004) | (0.004) | (0.005) |
| ln(other donor health spending per capita, baseline) |  |  | 0.0218*** |  |  |
|  |  |  | (0.005) |  |  |
| ln(other donor health spending per capita, yearly) |  |  |  | -0.0344*** | -0.0336*** |
|  |  |  |  | (0.004) | (0.004) |
| ln(domestic health spending per capita, baseline) |  |  | 0.0369* |  |  |
|  |  |  | (0.015) |  |  |
| ln(domestic health spending per capita, yearly) |  |  |  |  | -0.0397* |
|  |  |  |  |  | (0.017) |
| ln(BL GDP per capita) |  | -0.0217* | -0.0294* | -0.109*** | -0.0766*** |
|  |  | (0.011) | (0.015) | (0.015) | (0.019) |
| ln(BL HIV prevalence, % of population ages 15-49) |  | 0.00859*** | 0.0128*** | 0.0114*** | 0.0107*** |
|  |  | (0.002) | (0.002) | (0.003) | (0.003) |
| ln(BL life expectancy at birth) |  | -3.717*** | -3.126*** | -3.622*** | -3.656*** |
|  |  | (0.090) | (0.091) | (0.111) | (0.118) |
| ln(BL Urban population, %) |  | -0.0104 | -0.0508** | 0.000225 | 0.00972 |
|  |  | (0.016) | (0.016) | (0.019) | (0.021) |
| ln(BL School enrollment, secondary, % gross) |  | 0.0108 | 0.0946*** | 0.0264 | 0.0276 |
|  |  | (0.024) | (0.023) | (0.029) | (0.031) |
| ln(BL Fertility rate, births per woman) |  | -0.686*** | -0.379*** | -0.715*** | -0.726*** |
|  |  | (0.020) | (0.024) | (0.025) | (0.029) |
| Recipient of US HIV aid before 2004 (=1 if yes) |  | 0.0442** | -0.000476 | 0.0237 | 0.0214 |
|  |  | (0.017) | (0.016) | (0.021) | (0.022) |
| Constant | 1.992*** | 18.89*** | 15.48*** | 19.27*** | 19.27*** |
|  | (0.013) | (0.370) | (0.388) | (0.457) | (0.487) |
| Observations | 2544 | 2337 | 1982 | 1366 | 1183 |
| Adjusted R-squared | 0.118 | 0.589 | 0.598 | 0.619 | 0.631 |

Notes: Standard errors in parentheses; ***p < 0.001   **p < 0.01 * p< 0.05. Source: Our data came from four publicly available datasets: World Bank’s World Development Indicators; U.S. government’s foreignassistance.gov database; OECD Creditor Reporting System database; and the Institute of Health Metrics and Evaluation GBD Result’s Tool.

# Table N in S1 Table. Full model results for "Low intensity PEPFAR" group vs control: unlogged models

| **VARIABLES** | **Model 1** | **Model 2** | **Model 3** | **Model 4** | **Model 5** |
| --- | --- | --- | --- | --- | --- |
| Time variable (=1 post-2004) | -0.369* | -0.311* | -0.359** | 0.0158 | -1.021*** |
|  | (0.162) | (0.130) | (0.117) | (0.255) | (0.251) |
| Intervention (=1 if PEPFAR) | 2.362*** | 1.369*** | 0.769*** | 1.912*** | 1.172** |
|  | (0.199) | (0.207) | (0.187) | (0.433) | (0.405) |
| Interaction term (PEPFAR impact estimate) | -1.247*** | -1.329*** | -1.281*** | -1.208** | -0.527 |
|  | (0.277) | (0.215) | (0.193) | (0.422) | (0.395) |
| Country income level (=1 if middle income) |  | 0.913*** | 1.319*** | 1.767*** | 1.755*** |
|  |  | (0.238) | (0.219) | (0.307) | (0.301) |
| BL Population |  | -1.48e-09*** | -9.84e-10*** | -1.41e-09*** | -1.28e-09** |
|  |  | (0.000) | (0.000) | (0.000) | (0.000) |
| Other donor health spending per capita (baseline) |  |  | -0.0174*** |  |  |
|  |  |  | (0.002) |  |  |
| Other donor health spending per capita, yearly |  |  |  | -0.0115** | -0.00683* |
|  |  |  |  | (0.004) | (0.003) |
| Domestic health spending per capita (baseline) |  |  | 0.00761*** |  |  |
|  |  |  | (0.000) |  |  |
| Domestic health spending per capita, yearly |  |  |  |  | 0.00331*** |
|  |  |  |  |  | (0.000) |
| BL GDP per capita |  | 0.0000675*** | -0.000130*** | 0.0000407** | -0.0000467*** |
|  |  | (0.000) | (0.000) | (0.000) | (0.000) |
| BL HIV prevalence (% of population ages 15-49) |  | -0.301*** | -0.351*** | -0.305** | -0.280** |
|  |  | (0.080) | (0.072) | (0.101) | (0.099) |
| BL life expectancy at birth |  | -0.598*** | -0.678*** | -0.600*** | -0.638*** |
|  |  | (0.020) | (0.018) | (0.025) | (0.024) |
| BL Urban population (%) |  | -0.00619 | -0.0329*** | -0.00703 | -0.0225*** |
|  |  | (0.004) | (0.003) | (0.005) | (0.005) |
| BL School enrollment, secondary (% gross) |  | -0.00544 | -0.00316 | 0.000987 | 0.00101 |
|  |  | (0.004) | (0.004) | (0.005) | (0.005) |
| BL Fertility rate (births per woman) |  | -1.654*** | -1.335*** | -1.877*** | -1.562*** |
|  |  | (0.077) | (0.070) | (0.098) | (0.098) |
| Recipient of US HIV aid before 2004 (=1 if yes) |  | -0.910*** | -0.0523 | -1.274*** | -0.619* |
|  |  | (0.206) | (0.189) | (0.261) | (0.258) |
| Constant | 7.848*** | 53.92*** | 58.25*** | 53.52*** | 55.62*** |
|  | (0.116) | (1.488) | (1.348) | (1.905) | (1.858) |
| Observations | 2544 | 2308 | 2279 | 1349 | 1168 |
| Adjusted R-squared | 0.076 | 0.473 | 0.585 | 0.442 | 0.545 |

Notes: Standard errors in parentheses; ***p < 0.001   **p < 0.01 * p< 0.05. Source: Our data came from four publicly available datasets: World Bank’s World Development Indicators; U.S. government’s foreignassistance.gov database; OECD Creditor Reporting System database; and the Institute of Health Metrics and Evaluation GBD Result’s Tool.

# Table O in S1 Table. Full model results for "Low intensity PEPFAR" group vs control: logged models

| **VARIABLES** | **Model 1** | **Model 2** | **Model 3** | **Model 4** | **Model 5** |
| --- | --- | --- | --- | --- | --- |
| Time variable (=1 post-2004) | -0.0592** | -0.0553*** | -0.0769*** | 0.0186 | -0.0159 |
|  | (0.018) | (0.012) | (0.013) | (0.025) | (0.026) |
| Intervention (=1 if PEPFAR) | 0.250*** | 0.0725*** | 0.0650*** | 0.160*** | 0.149*** |
|  | (0.023) | (0.020) | (0.019) | (0.041) | (0.041) |
| Interaction term (PEPFAR impact estimate) | -0.0929** | -0.0980*** | -0.0928*** | -0.118** | -0.102* |
|  | (0.032) | (0.020) | (0.020) | (0.040) | (0.040) |
| Country income level (=1 if middle income) |  | 0.103*** | 0.0529* | 0.198*** | 0.185*** |
|  |  | (0.023) | (0.021) | (0.029) | (0.031) |
| ln(BL Population) |  | -0.0127*** | 0.00479 | -0.0343*** | -0.0314*** |
|  |  | (0.003) | (0.004) | (0.004) | (0.004) |
| ln(other donor health spending per capita, baseline) |  |  | 0.0305*** |  |  |
|  |  |  | (0.005) |  |  |
| ln(other donor health spending per capita, yearly) |  |  |  | -0.0354*** | -0.0331*** |
|  |  |  |  | (0.004) | (0.005) |
| ln(domestic health spending per capita, baseline) |  |  | 0.143*** |  |  |
|  |  |  | (0.014) |  |  |
| ln(domestic health spending per capita, yearly) |  |  |  |  | 0.0690*** |
|  |  |  |  |  | (0.017) |
| ln(BL GDP per capita) |  | -0.0137 | -0.0871*** | -0.0836*** | -0.118*** |
|  |  | (0.011) | (0.015) | (0.015) | (0.019) |
| ln(BL HIV prevalence, % of population ages 15-49) |  | 0.0134*** | 0.0163*** | 0.0137*** | 0.0129*** |
|  |  | (0.002) | (0.002) | (0.003) | (0.003) |
| ln(BL life expectancy at birth) |  | -3.523*** | -3.118*** | -3.413*** | -3.643*** |
|  |  | (0.091) | (0.096) | (0.117) | (0.127) |
| ln(BL Urban population, %) |  | -0.103*** | -0.164*** | -0.0712** | -0.103*** |
|  |  | (0.018) | (0.018) | (0.023) | (0.025) |
| ln(BL School enrollment, secondary, % gross) |  | -0.116*** | -0.0406* | -0.0831*** | -0.0725** |
|  |  | (0.019) | (0.018) | (0.025) | (0.026) |
| ln(BL Fertility rate, births per woman) |  | -0.786*** | -0.476*** | -0.769*** | -0.734*** |
|  |  | (0.018) | (0.023) | (0.026) | (0.028) |
| Recipient of US HIV aid before 2004 (=1 if yes) |  | -0.0272 | 0.00802 | -0.0250 | -0.0168 |
|  |  | (0.020) | (0.019) | (0.025) | (0.027) |
| Constant | 1.992*** | 18.87*** | 16.34*** | 18.91*** | 19.81*** |
|  | (0.013) | (0.356) | (0.381) | (0.451) | (0.493) |
| Observations | 2544 | 2308 | 1953 | 1349 | 1168 |
| Adjusted R-squared | 0.075 | 0.624 | 0.638 | 0.642 | 0.660 |

Notes: Standard errors in parentheses; ***p < 0.001   **p < 0.01 * p< 0.05. Source: Our data came from four publicly available datasets: World Bank’s World Development Indicators; U.S. government’s foreignassistance.gov database; OECD Creditor Reporting System database; and the Institute of Health Metrics and Evaluation GBD Result’s Tool.
