# Supplementary material for: Assessing the impact of the president’s emergency plan for AIDS relief on all-cause mortality
Source: PLOS Glob Public Health. 2024 Jan 18;4(1):e0002467. doi: 10.1371/journal.pgph.0002467 (PMC10796053; doi:10.1371/journal.pgph.0002467)
Supplement: S1 Fig — Fig A in S1 Fig. Residuals from unlogged Model 4 on all PEPFAR countries. Fig B in S1 Fig. Residuals from logged Model 4 on all PEPFAR countries. Fig C in S1 Fig. Residuals from unlogged Model 4 on COP countries. Fig D in S1 Fig. Residuals from logged Model 4 on COP countries. Fig E in S1 Fig. Residuals from unlogged Model 5 on all PEPFAR countries. Fig F in S1 Fig. Residuals from logged Model 5 on all PEPFAR countries. Fig G in S1 Fig. Residuals from unlogged Model 5 on COP countries. Fig H in S1 Fig. Residuals from logged Model 5 on COP countries. (DOCX) [file pgph.0002467.s006.docx]

# S1 Fig. Test the normal distribution of residuals derived from logged and unlogged Model 4 and Model 5 - Figs A - H

# Fig A in S1 Fig. Residuals from unlogged Model 4 on all PEPFAR countries

By visual check:

By statistical test:

Shapiro-Wilk test (H0: the data follow a normal distribution): reject H0 with p=0.00

# Fig B in S1 Fig. Residuals from logged Model 4 on all PEPFAR countries

By visual check:

By statistical test:

Shapiro-Wilk test (H0: the data follow a normal distribution): reject H0 with p=0.00

# Fig C in S1 Fig. Residuals from unlogged Model 4 on COP countries

By visual check:

By statistical test:

Shapiro-Wilk test (H0: the data follow a normal distribution): reject H0 with p=0.00

# Fig D in S1 Fig. Residuals from logged Model 4 on COP countries

By visual check:

By statistical test:

Shapiro-Wilk test (H0: the data follow a normal distribution): reject H0 with p=0.00

# Fig E in S1 Fig. Residuals from unlogged Model 5 on all PEPFAR countries

By visual check:

By statistical test:

Shapiro-Wilk test (H0: the data follow a normal distribution): reject H0 with p=0.00

# Fig F in S1 Fig. Residuals from logged Model 5 on all PEPFAR countries

By visual check:

By statistical test:

Shapiro-Wilk test (H0: the data follow a normal distribution): reject H0 with p=0.00

# Fig G in S1 Fig. Residuals from unlogged Model 5 on COP countries

By visual check:

By statistical test:

Shapiro-Wilk test (H0: the data follow a normal distribution): reject H0 with p=0.00

# Fig H in S1 Fig. Residuals from logged Model 5 on COP countries

By visual check:

By statistical test:

Shapiro-Wilk test (H0: the data follow a normal distribution): reject H0 with p=0.00
