# Supplementary material for: Assessing the impact of the president’s emergency plan for AIDS relief on all-cause mortality
Source: PLOS Glob Public Health. 2024 Jan 18;4(1):e0002467. doi: 10.1371/journal.pgph.0002467 (PMC10796053; doi:10.1371/journal.pgph.0002467)

# S2 Fig. Test the parallel assumption of ACM by country cohort

We tested the parallel trend of ACM between all PEPFAR countries and the non-PEPFAR countries. To do this, we regressed ACM on three variables – a dummy variable indicating countries’ PEPFAR status, a year categorical variable (with year 1990 as the reference year), and an interaction between the two. The coefficient of the interaction term indicates the difference in ACM trend between the PEPFAR and non-PEPFAR countries. A statistically insignificant coefficient of the interaction term, where its 95% confidence interval cross 0, means that there is no statistically significant difference in ACM trend between the two groups, and therefore, the parallel assumption holds between all PEPFAR and the control countries. We also did this similar test for the group of COP countries, non-COP PEPFAR countries, high intensity PEPFAR countries, medium intensity PEPFAR countries, and low intensity PEPFAR countries. Our tests indicate that the parallel assumption holds for all country cohorts before 2004.

# Fig A in S2 Fig. All PEPFAR countries versus control countries


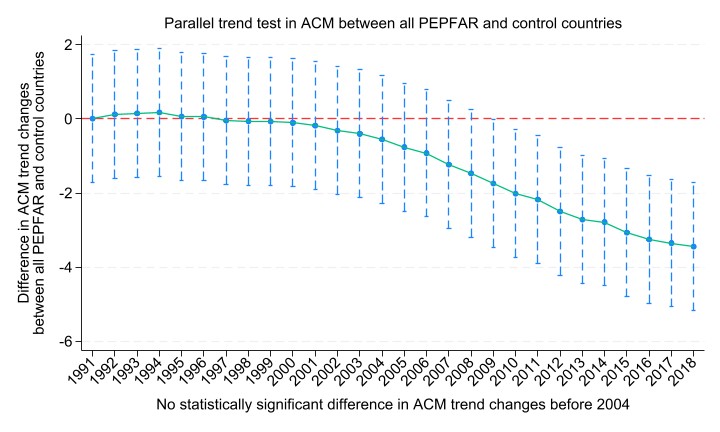


# Fig B in S2 Fig. COP countries versus control countries


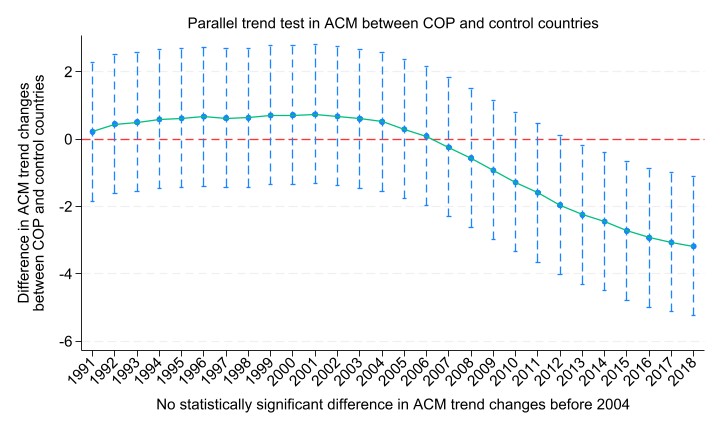


# Fig C in S2 Fig. Non-COP PEPFAR countries versus control countries


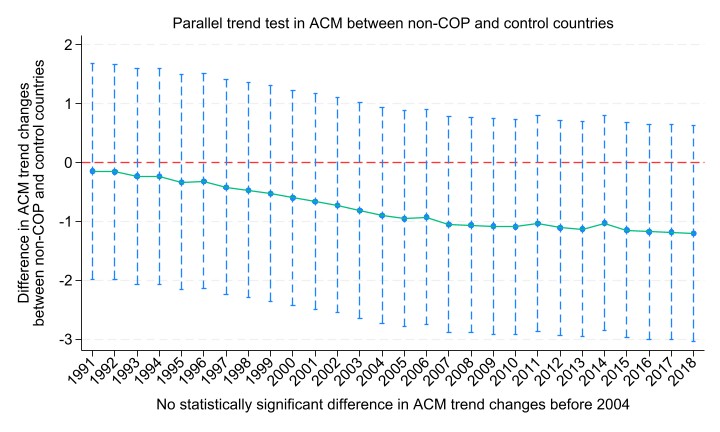


# Fig D in S2 Fig. High intensity countries versus control countries


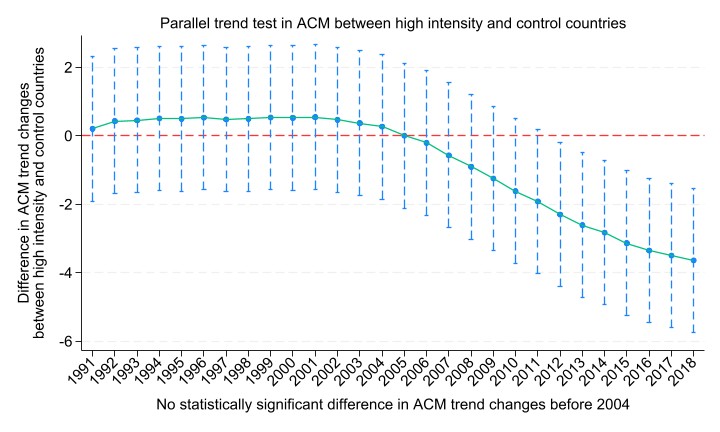


# Fig E in S2 Fig. Medium intensity countries versus control countries


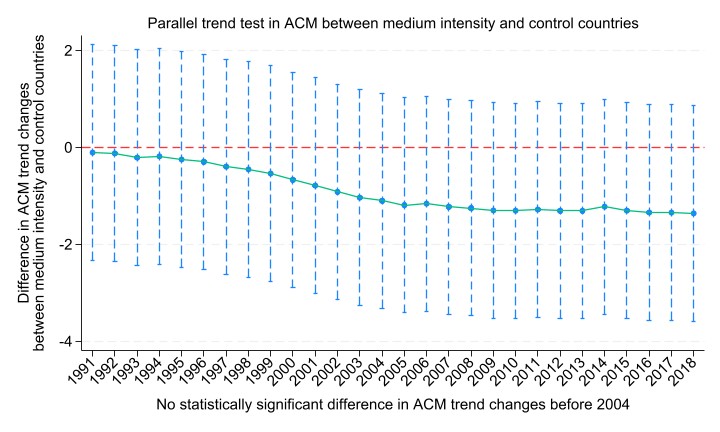


# Fig F in S2 Fig. Low intensity countries versus control countries


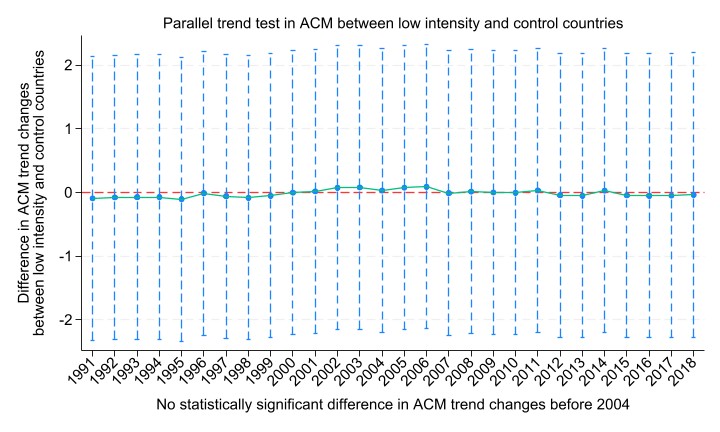

Supplement: S2 Fig — Fig A in S2 Fig. All PEPFAR countries versus control countries. Fig B in S2 Fig. COP countries versus control countries. Fig C in S2 Fig. Non-COP PEPFAR countries versus control countries. Fig D in S2 Fig. High intensity countries versus control countries. Fig E in S2 Fig. Medium intensity countries versus control countries. Fig F in S2 Fig. Low intensity countries versus control countries. (DOCX) [file pgph.0002467.s007.docx]
